# Feeding signals inhibit fluid‐satiation signals in the mouse lateral parabrachial nucleus to increase intake of highly palatable, caloric solutions

**DOI:** 10.1111/jnc.15991

**Published:** 2023-10-19

**Authors:** Connor M. Aitken, Janine C. M. Jaramillo, Warren Davis, Liam Brennan‐Xie, Stuart J. McDougall, Andrew J. Lawrence, Philip J. Ryan

**Affiliations:** ^1^ Florey Institute of Neuroscience & Mental Health University of Melbourne Parkville Victoria Australia; ^2^ Florey Department of Neuroscience & Mental Health University of Melbourne Parkville Victoria Australia

**Keywords:** AgRP, fluid satiation, oxytocin receptor, parabrachial nucleus

## Abstract

Chemogenetic activation of oxytocin receptor‐expressing neurons in the parabrachial nucleus (Oxtr^PBN^ neurons) acts as a satiation signal for water. In this research, we investigated the effect of activating Oxtr^PBN^ neurons on satiation for different types of fluids. Chemogenetic activation of Oxtr^PBN^ neurons in male and female transgenic *Oxtr* ^*Cre*^ mice robustly suppressed the rapid, initial (15‐min) intake of several solutions after dehydration: water, sucrose, ethanol and saccharin, but only slightly decreased intake of Ensure®, a highly caloric solution (1 kcal/mL; containing 3.72 g protein, 3.27 g fat, 13.42 g carbohydrates, and 1.01 g dietary fibre per 100 mL). Oxtr^PBN^ neuron activation also suppressed cumulative, longer‐term (2‐h) intake of lower caloric, less palatable solutions, but not highly caloric, palatable solutions. These results suggest that Oxtr^PBN^ neurons predominantly control initial fluid‐satiation responses after rehydration, but not longer‐term intake of highly caloric, palatable solutions. The suppression of fluid intake was not because of anxiogenesis, but because Oxtr^PBN^ neuron activation decreased anxiety‐like behaviour. To investigate the role of different PBN subdivisions on the intake of different solutions, we examined FOS as a proxy marker of PBN neuron activation. Different PBN subdivisions were activated by different solutions: the dorsolateral PBN similarly by all fluids; the external lateral PBN by caloric but not non‐caloric solutions; and the central lateral PBN primarily by highly palatable solutions, suggesting PBN subdivisions regulate different aspects of fluid intake. To explore the possible mechanisms underlying the minimal suppression of Ensure® after Oxtr^PBN^ neuron activation, we demonstrated in in vitro slice recordings that the feeding‐associated agouti‐related peptide (AgRP) inhibited Oxtr^PBN^ neuron firing in a concentration‐related manner, suggesting possible inhibition by feeding‐related neurocircuitry of fluid satiation neurocircuitry. Overall, this research suggests that although palatable beverages like sucrose‐ and ethanol‐containing beverages activate fluid satiation signals encoded by Oxtr^PBN^ neurons, these neurons can be inhibited by hunger‐related signals (agouti‐related peptide, AgRP), which may explain why these fluids are often consumed in excess of what is required for fluid satiation.

## Graphical abstract

Oxytocin receptor‐expressing neurons in the parabrachial nucleus (Oxtr^PBN^) act as a satiation signal for water. We found that activating these neurons reduced initial intake of sucrose, ethanol and saccharin after overnight dehydration. Our study showed that different parabrachial subdivisions regulate different aspects of fluid intake: the dorsolateral (dl) regulates intake of all fluids; the external lateral (el) regulates intake of high‐calorie solutions; and the central lateral (cl) regulates highly palatable solutions. We observed that hunger‐related signals (AgRP; agouti‐related peptide) inhibit Oxtr^PBN^ neurons, which might explain why high‐calorie solutions are often consumed in excess of what is required for fluid satiation.

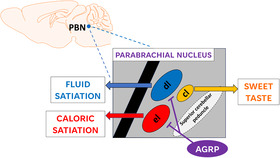

**Abbreviations**
2BC: Two bottle choice
aCSF: Artificial cerebrospinal fluid
AAV: Adeno‐associated virus
AgRP: Agouti‐related peptide
AgRP^ARC^
: Agouti‐related peptide‐expressing in the arcuate nucleus
AgRP^Cre^
: Knock‐in mouse line which has the Cre recombinase enzyme inserted downstream of the stop codon of the agouti‐related peptide gene
Ai14: Cre‐reporter mouse strain designed to express robust tdTomato fluorescence following Cre‐mediated recombination.
AP: Anteroposterior
ARC: Arcuate nucleus
Calca^Cre^
: Knock‐in mouse line which has the Cre recombinase enzyme inserted downstream of the stop codon of the calcitonin/calcitonin‐related polypeptide, alpha gene
CGRP: Calcitonin gene‐related peptide
CGRP^PBN^
: Calcitonin gene‐related peptide‐expressing in the parabrachial nucleus
cl: Central lateral
CNO: Clozapine‐N‐oxide
CPP: Conditioned place preference
DIO: Double‐floxed inverse open reading frame
dl: Dorsolateral
DREADD: Designer receptor exclusively activated by designer drugs
DV: Dorsoventral
EGTA: Egtazic acid, a chelating agent
el: External lateral
EPM: Elevated plus maze
hM_3_Dq: Stimulatory DREADD; modified form of the human M3 muscarinic receptor
ip: Intraperitoneally
LOF: Large open field
LPBN: Lateral parabrachial nucleus
MC3R: Melanocortin 3 receptor
MC4R: Melanocortin 4 receptor
ML: Mediolateral
MnPO: Median preoptic nucleus
MnPO^GLP1r^
: Glucagon‐like peptide‐1 receptor expressing in the median preoptic nucleus
*n*
: Number of mice
NDS: Normal donkey serum
Oxtr: Oxytocin receptor
Oxtr^Cre^
: Knock‐in mouse line which has the Cre recombinase enzyme inserted downstream of the stop codon of the oxytocin receptor gene
Oxtr^PBN^
: Oxytocin receptor‐expressing in the parabrachial nucleus
PBN: Parabrachial nucleus
PBS: Phosphate‐buffered saline
PBST: Phosphate‐buffered saline with addition of detergent, Triton X‐100
Pdyn: Prodynorphin
Pdyn^PBN^
: Prodynorphin‐expressing in the parabrachial nucleus
POMC: proopiomelanocortin
scp: Superior cerebellar peduncle
sctv: Ventral spinocerebellar tract
s.e.m.: Standard error of the mean
SFO^GLP1r^
: Glucagon‐like peptide‐1 receptor‐expressing in the subfornical organ
TdT: tdTomato
v/v: Volume/volume
w/v: Weight/volume

## 1 INTRODUCTION

Satiation is a vital signal preventing overconsumption of food and fluid and is essential for maintaining energy and fluid homeostasis (Morton et al., 2014; Ryan, 2018). A key brain region regulating satiation is the lateral parabrachial nucleus (LPBN) in the hindbrain (Palmiter, 2018), which comprises several subdivisions, including the dorsolateral (dl) LPBN which controls fluid satiation, and the external lateral (el) LPBN which primarily controls food satiation (Carter et al., 2013; Ryan et al., 2017). The major phenotype of LPBN neurons is excitatory, glutamatergic neurons (Pauli et al., 2022); however, there are several markers which have been identified for neurons within the LPBN subdivisions, including Oxtr (oxytocin receptor) predominantly for dl LPBN neurons and CGRP (calcitonin gene‐related peptide) for el LPBN neurons. Chemogenetically activating Oxtr^PBN^ neurons suppresses fluid intake, whereas activating CGRP^PBN^ neurons primarily suppresses food intake (Campos et al., 2016, 2018; Carter et al., 2013; Ryan et al., 2017).

Activating Oxtr^PBN^ neurons decreased intake of water, but not highly caloric, palatable fluids like 1 kcal/mL Ensure® (Ryan et al., 2017), suggesting LPBN neurons might differentiate solutions based on their caloric content and/or palatability; however, the underlying mechanism for this differentiation is unknown. This a vital research area, given that highly caloric, palatable drinks such as sodas (soft drinks) and alcoholic beverages are key contributors to the obesity epidemic (Schulze et al., 2004; Shelton & Knott, 2014) and are often advertised for their thirst‐quenching properties. Using *Oxtr* ^*Cre*^ mice, we investigated the role of dl LPBN neurons in satiation for different fluids, including caloric solutions (Ensure®, ethanol, sucrose), and non‐caloric solutions (saccharin, saline), as well as a mechanism to explain how different solutions are distinguished.

## 2 METHODS

### 2.1 Mice

Experiments were conducted with the approval of the Florey Institute of Neuroscience and Mental Health Animal Ethics Committee (Ethics numbers 17‐069‐FINMH and 20‐036‐FINMH) and according to ethical guidelines issued by the National Health and Medical Research Council of Australia. The following mouse lines were used in these experiments: C57Bl/6J (wild type; RRID:MGI:2159769; Animal Resource Centre, Murdoch, WA, Australia), *Oxtr* ^*Cre*^ (RRID:IMSR_JAX:030543) and *Calca* ^*Cre*^ mice (RRID:IMSR_JAX:033168) (provided by Richard D. Palmiter, University of Washington, Seattle, WA, USA), which were bred onto a C57Bl/6J background. Both heterozygotes and homozygotes were used because no differences in behaviour or expression were observed. The *Oxtr* ^*Cre*^ mice were crossed to a tdTomato‐reporter mouse line: *Gt(ROSA)26Sor* ^*tm14(CAG‐tdTomato)Hze*^/J mice (Ai14; RRID:IMSR_JAX:007914; Allen Institute, Seattle, WA, USA). We have previously confirmed adult expression of Oxtr by in situ hybridisation in *Oxtr* ^*Cre*^::Ai14 mice (Ryan et al., 2017).

Mice in each litter were arbitrarily assigned to either experimental or control groups. No formal randomisation procedure was performed to allocate subjects in the study; however, groups were counterbalanced for age and sex. Unless otherwise stated, fluid experiments were performed on at least two cohorts of both adult male and female mice (8–26 weeks old at the start of experimentation), given that no difference was observed in fluid intake between sexes (Ryan et al., 2017). We also found no significant difference in fluid intake at different ages; for example, a comparison of two cohorts with different ages (Cohort 1: 9.2 ± 0.1 weeks and Cohort 2: 24.3 ± 0.8 weeks) found no significant difference in total fluid intake. At 15 min, Cohort 1 versus 2: 0.6 ± 0.1 mL versus 0.6 ± 0.1 mL; *t*(9) = 0.33; *p* = 0.75; and at 2 h, Cohort 1 versus 2: 1.7 ± 0.3 mL versus 1.9 ± 0.2 mL; 7(9) = 0.41; *p* = 0.69. Refer to Table S1 for a summary of the sexes and ages of the different mouse cohorts in the fluid experiments.

Before surgery or experimentation, mice were group housed and maintained on a Barastoc rodent diet (Product code: 102093; Ridley Co., Melbourne, VIC, Australia) with water available ad libitum in a 12‐h light:dark cycle at 18–24°C; humidity 40%–70%. Mice were at least 7 weeks old before surgery. Following surgery or during experimentation, mice were single housed.

### 2.2 Virus production

AAV2‐hSyn‐DIO‐hM_3_D(Gq):mCherry was a gift from Bryan L. Roth (Addgene viral prep # 44361‐AAV2; http://n2t.net/addgene:44361; Addgene, Watertown, MA, USA; RRID:Addgene_44361) (Krashes et al., 2011); titre: ≥ 6 × 10^12^ vg/mL. AAV1/2‐CAG‐FLEX‐tdTomato and was prepared by Sharon L. Layfield and Ross A. D. Bathgate (Florey Institute of Neuroscience and Mental Health, Parkville, VIC, Australia); titre: 8.7 × 10^11^/mL.

### 2.3 Stereotaxic surgery

Mice were anaesthetised with isoflurane in air (5% v/v induction; 1%–2% v/v maintenance) (IsoFlo®; Zoetis Australia Pty Ltd, Rhodes, NSW, Australia), placed in a stereotaxic frame (Stoelting, Wood Dale, IL, USA; catalogue number: 51730) and injected with meloxicam (1–3 mg/kg i.p.; product code: MELOX2; Troy Laboratories, Glendenning, NSW, Australia) for analgesia. Stereotaxic coordinates were normalised for the anterior–posterior plane by a correction factor (*F* = distance between bregma and lambda/4.21) (Paxinos & Franklin, 2013). Viruses were injected (500 nL bilaterally) via glass pipettes using the Nanoject III Auto‐Nanoliter Injector (Drummond Scientific Company, Broomall, PA, USA; catalogue number: 3‐000‐207). Viruses were injected according to the following coordinates: PBN (AP: −5.2 mm; ML: ±1.35 mm; DV: −3.1 mm). This infusion site (which is not directly at the site of the Cre‐expressing neurons) and this volume have previously been used for parabrachial nucleus injections, and have been found to improve the accuracy of injecting into the lateral PBN between the superior cerebellar peduncle (scp) and the lateral wall of the pons, and improved the success rate of injections (Campos et al., 2016; Carter et al., 2013; Ryan et al., 2017). Also, the presence of the scp fortuitously limits the spread of virus from the PBN region, thus preventing unintended transduction of other nearly brain regions. Virus was injected over 4 min, with 2 min wait time prior to removal of the injector. Postoperatively, mice recovered in heated chambers with constant observation until they were mobile. Mash feed, consisting of rodent diet mixed in warm water, was offered post‐operatively to aid recovery. Mice were given at least 2 weeks to recover before experimentation to allow for adequate viral transfection (Ryan et al., 2017).

### 2.4 Tissue processing

Following experimentation, mice were anaesthetised using Lethabarb 80 mg/kg i.p. (pentobarbitone sodium 325.73 g/L; product code: LETH; Virbac (Australia) Pty Ltd, Milperra, NSW, Australia) and perfused transcardially with phosphate‐buffered saline (PBS) followed by 4% w/v paraformaldehyde (Sigma‐Aldrich, Burlington, MA, USA; catalogue number: 158127‐100G) dissolved in PBS to transform it into formaldehyde. Brains were removed and placed in the formaldehyde solution overnight, then cryoprotected in 30% w/v sucrose in PBS, then Scigen™ O.C.T. Compound (Scigen, Paramount, CA, USA; catalogue number: 4586‐1) and kept at −80°C before processing.

Coronal brain sections (30‐μm) were cut on a Leica CM1900 cryostat (Leica Microsystems, Wetzlar, Germany). For all FOS studies, every third section was collected (90 μm apart) for quantification. To confirm viral hits, every sixth brain section was mounted onto glass slides (Superfrost® Plus; Thermo Fisher Scientific, Waltham, MA, USA) and coverslipped with Fronine safety mount No. 4 (Thermo Fisher Scientific; catalogue number: FNNII068), and remaining sections were collected in PBS with 1% w/v azide (VWR, Radnor, PA, USA; catalogue number: BDH7465‐2) for storage.

### 2.5 Immunohistochemistry

#### 2.5.1 FOS and CGRP studies

Immunohistochemistry was performed on free‐floating sections. For FOS in the PBN, we used *Oxtr* ^*Cre*^::Ai14 mice. Sections underwent three 5‐min washes in PBST (phosphate‐buffered solution with 0.1% v/v Triton X‐114 (Sigma‐Aldrich; catalogue number: X114)) and were placed in a blocking solution (PBST with 3% v/v normal donkey serum [NDS]; Jackson ImmunoResearch, West Grove, PA, USA; catalogue number: 017‐000‐121; RRID:AB_2337258) for 1 h at room temperature (20–22°C) to reduce nonspecific binding that may result from applying secondary antibody to the tissue. Sections were then incubated with rabbit anti‐c‐FOS (1:1000; Santa Cruz Biotechnology Inc, Dallas, TX, USA; catalogue number: sc‐52; RRID:AB_2106783) in 3% v/v NDS in PBST overnight at room temperature. After three 5‐min washes in PBST, the sections were incubated in the dark with donkey anti‐rabbit IgG (H+L) Alexa Fluor® 488 (1:500; Thermo Fisher Scientific; catalogue number: A‐11055; RRID:AB_2534102) in 3% v/v NDS in PBST for 1 h at room temperature. The sections were then washed three times (5 min each) in PBS, before being mounted onto glass slides and cover‐slipped using safety mount.

For colocalisation of CGRP and FOS in *Oxtr* ^*Cre*^::Ai14 mice, the primary antibodies were goat anti‐CGRP (1:500; Abcam Cambridge, UK; catalogue number: ab36001; RRID:AB_725807) and rabbit anti‐c‐FOS (1:1000; Santa Cruz Biotechnology; catalogue number: sc‐52; RRID:AB_2106783), and the secondary antibodies were donkey anti‐goat IgG (H+L) Alexa Fluor® 488 (1:500; Thermo Fisher Scientific; catalogue number: A‐11055; RRID:AB_2534102) and donkey anti‐rabbit IgG (H+L) Alexa Fluor® 647 (1:500; Molecular Probes (Invitrogen), now part of Thermo Fisher Consolidation; catalogue number: A‐31573; RRID:AB_2536183).

#### 2.5.2 AgRP fibres

To assess AgRP fibres in the parabrachial regions, the primary antibody was human AgRP(83‐132 amide) (rabbit polyclonal 1:200; Phoenix Pharmaceuticals, Burlingame, CA, USA; catalogue number H‐003‐53; RRID:AB_2313908) and the secondary antibody was Donkey anti‐rabbit IgG (H+L) Alex Fluor® 488 (1:500; Thermo Fisher Scientific; catalogue number: A‐11055; RRID:AB_2534102).

#### 2.5.3 Viral targeting

To assess targeting and neuronal activity in hM3Dq‐injected mice, the primary antibodies were Living Colours® rabbit anti‐dsRed (1:2000; Takara Bio Clontech, Kusatsu, Shiga, Japan; catalogue number: 632496; RRID:AB_10013483) to amplify the fluorescent protein signal (used only for hM_3_Dq‐injected *Oxtr* ^*Cre*^ mice) and goat anti‐c‐FOS (1:1000; Santa Cruz Biotechnology; catalogue number: sc‐52‐G; RRID:AB_2629503). Secondary antibodies were donkey anti‐goat IgG (H+L) Alexa Fluor® 488 (1:500; Thermo Fisher Scientific; catalogue number: A‐11055; RRID:AB_2534102) and donkey anti‐rabbit IgG (H+L) Alexa Fluor® 594 (1:500; Molecular Probes (Invitrogen), now part of Thermo Fisher Consolidation; catalogue number: A‐21207; RRID:AB_141637). The amplification step was not necessary for tdTomato‐injected or *Calca* ^*Cre*^ mice, so only FOS was investigated.

### 2.6 Microscopy

Brain sections were imaged on a Leica SP8 confocal microscope (Leica Microsystems). Image analysis and optimisation were conducted using two programs, ImageJ and Leica's proprietary software, LAS‐X. Minimal processing of images for brightness and contrast was performed as necessary to ensure optimal representation of the parabrachial nucleus and neuronal expression.

Following imaging for targeting, any mouse whose targeted injection site was outside the PBN was excluded from experimental analysis. Both bilateral and unilateral hits were included for activation experiments.

### 2.7 FOS experiments

For FOS studies, *Oxtr* ^*Cre*^::Ai14 mice were single‐housed. For fluid intake experiments, we designed 20 mL customised tubes (15 mL Falcon tube adjoined to pipette and sipper with ball‐bearing) which were angled at ~30° into the mouse's cage. Mice were habituated for at least 3 days to a two‐bottle choice paradigm, with water in one tube and an experimental solution in the other. The experimental solutions were: 1 kcal/mL Ensure® (vanilla powder; Abbott Laboratories, Abbott Park, IL, USA), 10% v/v ethanol (Chem‐Supply, Port Adelaide, Australia; catalogue number: EL043‐5L‐P), 15% w/v sucrose (Chem‐Supply, catalogue number: SA030‐500G), 0.1% w/v saccharin (Sigma‐Aldrich; catalogue number: 109185‐250G), 0.15 M NaCl (Chem‐Supply; catalogue number: SA046‐500G). Fluids were removed for 24 h, then either water or the experimental solution was reintroduced; mice were perfused 2 h after fluid return. During the experiment, mice had access to ad libitum food intake. Prior to removal of fluids, the preferences for each experimental fluid versus paired water were as follows (mean ± s.e.m.): ethanol 42.7 ± 8.9%; sucrose 88.5 ± 9.2%; saccharin 92.3 ± 1.4%; Ensure 97.0 ± 0.5%; saline 23.0 ± 2.9%.

#### 2.7.1 Fasting and dehydration studies

For fasting, mice had both chow and Ensure® removed for 24 h; for dehydration, mice had both water and Ensure® removed for 24 h. Ensure® was returned 24 h later, with mice perfused 2 h after Ensure® return.

Quantification of FOS immunoreactivity was performed on seven 30‐μm coronal brain sections each 90 μm apart, ranging from bregma −5.0 to −5.7 mm (encompassing the major portion of the PBN where *Oxtr* is expressed). The investigator who quantified FOS immunoreactivity was blinded to the identity of the fluid. Given that every third section was counted, an estimate of total counts was made by multiplying by 3.

### 2.8 Fluid intake experiments

Mice were single housed after surgery until recovery. After 2–3 weeks recovery from surgery, mice were handled and habituated to vehicle injections for several days. Prior to experimentation, mice were injected with CNO (3 mg/kg ip; Advanced Molecular Technologies Pty Ltd, AMTA056‐CO16, Scoresby, VIC, Australia; catalogue number: AMTA065‐CH17‐500) (Jendryka et al., 2019) and examined for their 2‐h water intake after 24‐h dehydration. If hM_3_Dq‐injected mice drank >1 mL water over 2 h, suggestive of incorrect targeting (Ryan et al., 2017), they were excluded from further experimentation (verified in 2 mice post‐mortem).

Mice were given 2–3 days to habituate to the 2‐bottle choice paradigm (water and Ensure®/sucrose/ethanol/saccharin/saline). During experimentation, tubes were weighed at 15‐min intervals over 2 h. To account for leakage, we weighed an extra set of tubes which had been placed in an empty mouse box and subtracted any leakage from the results (typically 0.1–0.3 mL over 2 h). We consistently used the left side for water and the right side for the experimental fluid, which was useful for comparing intake of experimental fluids at higher versus lower concentrations (i.e. placing the higher concentration fluids on the right side of the cage at the start of 2BC experiments meant that mice tended to drink more from that side, including in later experiments at lower concentrations which otherwise might be difficult for the mice to distinguish from water); however, we note that we would be unable to accurately assess fluid preference (Tordoff & Bachmanov, 2002).

At the end of experimentation, mice were killed by transcardial perfusion 2 h after CNO (3 mg/kg i.p.) injection, as described, and targeting was validated by immunohistochemistry for reporter and FOS immunoreactivity. For correlation experiments, both unilateral and bilateral hits were included (the right and left sides were averaged for the bilateral hits).

### 2.9 Anxiety‐like behaviour, locomotion and conditioned place preference

#### 2.9.1 Locomotor cell

Eight locomotor cells were used for this study, each consisting of a transparent box approximately 30 cm^2^ with infrared technology to detect mouse movement (Med Associates Inc., Fairfax, VT, USA), and a lid to prevent mice from jumping out of the cell. All test mice were brought into the locomotor cell room 1 h prior to testing for habituation. Soft lighting from light bars above the cells was used to provide a consistent and less stressful level of illumination (~30–50 lux). *Oxtr* ^*Cre*^ mice were injected with CNO (3 mg/kg) or vehicle 30 min prior to testing. Testing consisted of 1 h free exploration of the locomotor cell with one mouse in each chamber.

#### 2.9.2 Elevated plus maze

The same cohort of mice underwent the elevated plus maze (EPM) test approx. 3 days later, which was a plus‐shaped structure raised 50 cm off the ground with two open arms and two closed arms. A camera attached to the roof was then focused on the field and configured with ‘About Activity Monitor’ (version 7.0.5.10, Med Associates Inc. 2015). The mouse was placed in the centre zone of the EPM and activity was monitored for 10 min. This experiment was performed between 09:30 and 11:30, and the hM_3_Dq‐ and tdT‐injected mice were alternated.

#### 2.9.3 Large open field

The same cohort of mice underwent the large open field (LOF) test 10 days later. The open field consisted of a 50 cm high metal sheet forming a circle of 1.5‐m diameter with light set to ~1000 lux. The linoleum floor of the room was cleaned and left uncovered for the experiment. The camera and software used were the same as for the EPM except the zones were altered. Mice were habituated to the room for 30 min before the first test and injected with CNO (3 mg/kg i.p.) 30 min prior to testing. Mice were placed in the middle of the field and left to roam freely for 10 min. This experiment was performed between 09:30 and 11:30, and the hM_3_Dq‐ and tdT‐injected mice were alternated, and the order of mice was reversed from the elevated plus maze experiment.

#### 2.9.4 Conditioned place preference

Four identical CPP apparatuses consisting of three compartments were used, which included two main larger compartments separated by a centre smaller compartment (Harvard Apparatus, Holliston, MA, USA). The two larger compartments had differences in visual and tactile properties (one had swirls on the walls and a soft floor; the other had stripes on the walls and a rippled floor). The apparatuses each had a roof containing separate lighting for each compartment (~75 lux in each). The experiment was performed over 10 days and all sessions were 30 min, with mice being habituated to the room for 30 min prior. On the first day, mice were habituated to the compartments with no restrictions on their movement. Following this were 8 days of conditioning where mice were given intraperitoneal injections of either saline or CNO (3 mg/kg) in a counterbalanced manner and placed in one of the two larger compartments. On the 10th day, mice were allowed free movement between the chambers. Their time spent in each chamber was analysed and compared with the first day of free roaming.

### 2.10 Electrophysiology

#### 2.10.1 Whole‐cell recordings

*Oxtr* ^*Cre*^::Ai14 mice (*n* = 12, 5 female; 7 male, 18–38 g, P37–179) underwent lethal overdose with 5% v/v isoflurane. They were monitored to determine that they were unresponsive to paw pinch and lacked respiratory signs to ensure surgical depth anaesthesia had been reached, then the pons and brainstem removed, blocked and rapidly cooled in artificial cerebrospinal fluid (aCSF, 2°C, containing (in mM): NaCl, 125; KCl, 3; KH_2_PO_4_, 1.2; MgSO_4_, 1.2; NaHCO_3_, 25; dextrose, 10; CaCl_2_, 2; (300 mOsmol)). Coronal 200 μm slices of the pons incorporating the PBN were cut on a vibratome (VT1200s; Leica Microsystems). The slices were continuously perfused in aCSF solution, bubbled with 95% O_2,_ 5% CO_2,_ at 32°C. Recording pipettes (3.0–5.3 MΩ) contained internal solution (in mM): NaCl, 6; NaOH, 4; KOH, potassium gluconate, 130; EGTA, 11; CaCl_2_, 1; HEPES, 10; MgCl_2_, 1; 0.1% biocytin (pH 7.3, 290 mOsmol). Pipettes were visually guided to tdTomato‐expressing PBN neurons using a fixed stage scope (Axio Examiner; Zeiss, Thornwood, NY, USA) and camera (*Rolera EM‐C2*; Q‐Imaging, Surrey, BC, Canada). Whole‐cell recordings were made in current clamp mode (MultiClamp 700B and pClamp 10.3; Molecular Devices, San Jose, CA, USA). Signals were sampled at 20 kHz and filtered at 10 kHz. Liquid junction potentials were not corrected (−6.6 mV at 32°C until post). AGRP (10, 100 or 1000 nM; Agouti‐related peptide (AgRP) (83–132) (Human); Phoenix Pharmaceuticals; catalogue number: 003‐53) was superfused onto the slice in aCSF at 2.5 mL/min.

### 2.11 Data analysis and statistics

Data analysis and generation of histograms were performed using GraphPad Prism Version 8.3.0 for Windows (GraphPad Software, La Jolla, CA, USA). Results are expressed as mean ± s.e.m. Statistical significance was determined by different tests appropriate for each data set: for comparing the means of two groups, unpaired two‐tailed Student's *t*‐test; for comparing fluid intake at different time‐points, a repeated measures two‐way ANOVA with Sidak's post hoc tests; for correlation studies, a Pearson product–moment correlation. We also tested for equality of variance (Levene's test of equality of variance). Equality of variance was assessed using the Brown‐Forsythe test, assuming any differences are because of variability. Data distribution was tested using D'Agostino‐Pearson normality test (omnibus K2) and Shapiro–Wilk test (Royston (1)). For changes in membrane potential, 60‐s periods were averaged prior to AgRP exposure and at peak AgRP response and tested via paired *t*‐tests at each AgRP concentration. Similarly for input‐frequency curves, step protocols were run prior to, and at peak AgRP response, in each Oxtr^PBN^ neuron and tested with a repeated measures two‐way ANOVA with Bonferroni post hoc tests. ****p < 0.0001; ***p < 0.001; **p < 0.01; *p < 0.05.

A power analysis was initially performed for an effective sample size using http://powerandsamplesize.com/. Based on previous experiments (Ryan et al., 2017), and a pilot study investigating 0.6 kcal/mL Ensure® intake, we calculated means of 1.8 (Ensure® intake) and 1.1 (water intake) with a standard deviation of 0.4. We estimated means were 1.5‐fold different with a two‐tailed Student's *t*‐test and assumed a significance level of 0.05 and power size of 0.8, so calculated a sample size of 6 per group for DREADD/WT studies (actual group size did not always reflect sample size as we usually injected 1–3 additional mice to allow for missed targeting and occasionally excluded mice at the end of experimentation because of missed targeting). We based sample size for FOS experiments on previous studies by Ryan et al. (2017), with means of 25 (approx. percentage of FOS‐immunoreactive neurons after fluid return) and 5 (approximate percentage of FOS‐immunoreactive neurons in fluid‐deprived mice) and a standard deviation of 10. We used the same assumptions as above (1.5‐fold different, 0.8 power size) and calculated a sample size of 4.

## 3 RESULTS

### 3.1 Activation of Oxtr^PBN^ neurons decreased Ensure® intake in a concentration‐related manner

**FIGURE 1:**
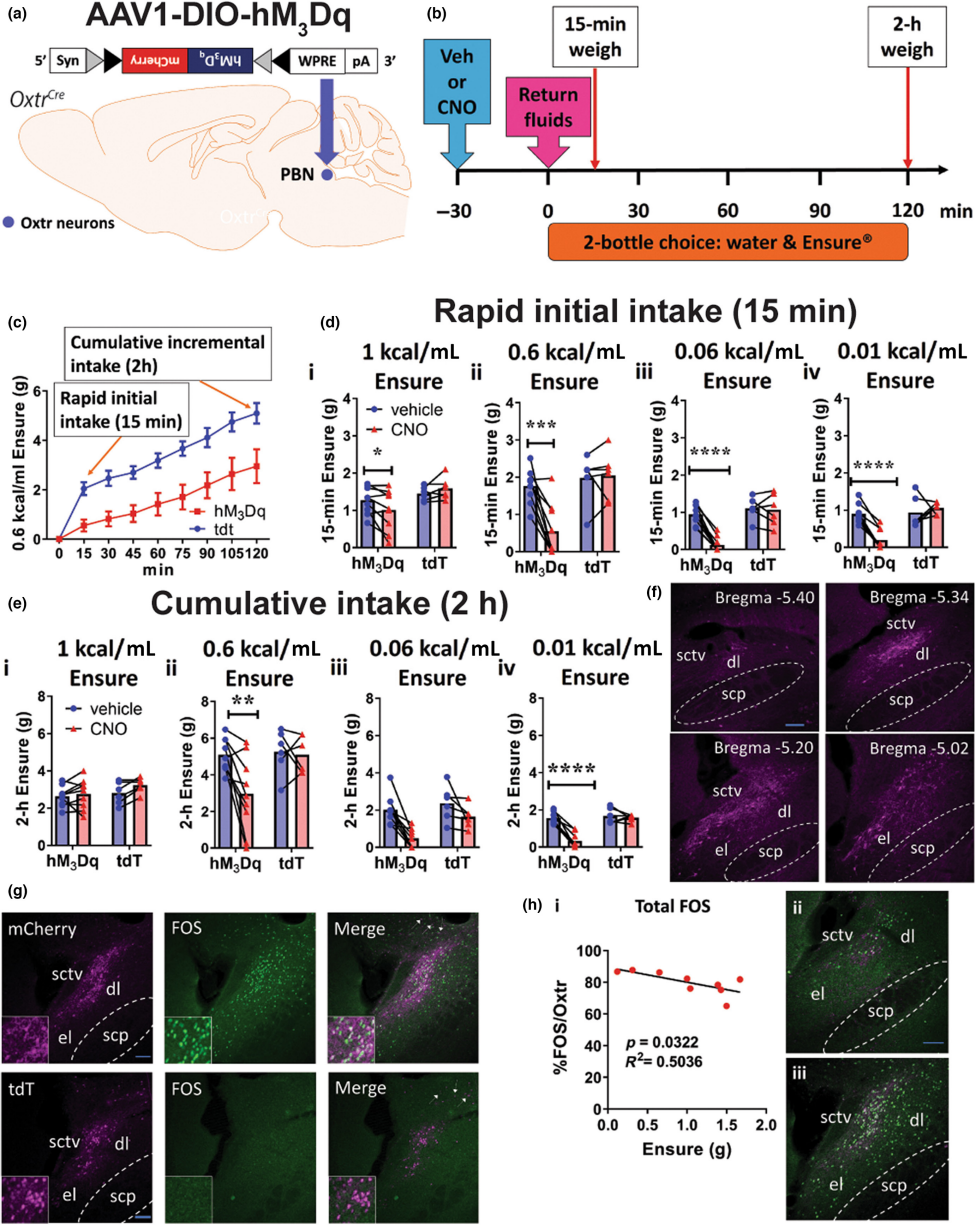
Activation of Oxtr^PBN^ neurons decreased intake of Ensure® in a concentration‐related manner. (a) Injection of AAV‐DIO‐hM_3_Dq:mCherry in Oxtr^PBN^ neurons. Grey and black triangles denote *loxP* and *lox2722* sites, respectively. (b) After habituation to the two‐bottle choice paradigm over 2–3 days, mice were injected with either vehicle or 3 mg/kg CNO ip; then water and Ensure® bottles were returned 30 min later (after overnight dehydration); and bottles were weighed at 15‐min and 2‐h timepoints. These experiments were repeated with Ensure® at several different concentrations: 1, 0.6, 0.06 and 0.01 kcal/mL. (c) Example intake profile of 0.6 kcal/mL Ensure® for hM_3_Dq‐ and tdT‐injected mice after 3 mg/kg CNO injection, which includes 15‐min (rapid) and 2‐h (cumulative) intake of fluids. Fluid intake was measured for the initial 15 min of fluid return, which was generally substantial following dehydration in control tdT‐injected mice; and continued to increase for both hM_3_Dq‐ and tdT‐injected mice over 2‐h (cumulative intake). (d) Acute Oxtr^PBN^ stimulation decreased 15‐min rapid intake of (i) 1 kcal/mL Ensure®; (ii) 0.6 kcal/mL Ensure®; (iii) 0.06 kcal/mL Ensure®; (iv) 0.01 kcal/mL Ensure®. (e) Oxtr^PBN^ activation decreased 2‐h cumulative intake of Ensure® in a concentration‐related manner. (i) No significant effect of Oxtr^PBN^ activation was observed for cumulative intake of 1 kcal/mL Ensure®. (ii–iv) Oxtr^PBN^ activation suppressed cumulative intake of lower concentrations of Ensure® (0.6 and 0.01 kcal/mL), but had no significant effect on 0.06 kcal/mL Ensure® or paired water intake. Data expressed as mean ± s.e.m.; *n* = 9 hM_3_Dq, 6 tdTomato; ****p < 0.0001; ***p < 0.001; *p < 0.05. (f) Example images of viral spread through PBN from Bregma −4.96 to −5.40 mm. Scale bar represents 100 μm. (g) Validation of hM3Dq‐ and tdTomato‐expressing viruses in the PBN following CNO injection. Following DREADD studies experimentation, mice were injected with CNO (3 mg/kg ip) 2 h prior to perfusion and targeting verified by examining tagged fluorescence and FOS immunoreactivity. Scale bar represents 100 μm. Arrows show some mild fluorescence in the adjacent cerebellar region. (h) Inverse correlation of % FOS immunoreactivity in Oxtr^PBN^ neurons and 15‐min 1 kcal/mL Ensure® intake following hM3Dq activation. (i) An inverse correlation was observed between Ensure® intake and % FOS immunoreactivity following hM3Dq activation for FOS immunoreactivity in the lateral PBN. (ii–iii) Representative images demonstrating comparison between low and high immunoreactivity of FOS in the LPBN following hM3Dq activation. *n* = 7 (bilateral); 2 (unilateral); hM_3_Dq mice. Scale bar represents 100 μm. dl, dorsolateral; el, external lateral; scp, superior cerebellar peduncle; sctv, ventral spinocerebellar tract.

Given that Oxtr^PBN^ neuron activation suppressed water but not highly caloric (1 kcal/mL) Ensure® solution intake (Ryan et al., 2017), we investigated the effect of Oxtr^PBN^ neuron activation on different concentrations of Ensure®. We injected an adeno‐associated virus (AAV) carrying the Cre‐dependent hM_3_Dq:mCherry (AAV‐DIO‐hM_3_Dq:mCherry) bilaterally into the PBN of *Oxtr* ^*Cre*^ mice (Figure 1a). As a control, we injected a Cre‐dependent virus containing a red fluorescent marker, AAV‐DIO‐tdTomato. For 4 h/day, mice had access to a two‐bottle choice of water versus Ensure® at 0.01, 0.06, 0.6 and 1 kcal/mL (N.B. 1 kcal/mL is manufacturer's recommended dose for human consumption; 0.6 kcal/mL was chosen because it is approx. isocaloric to 15% w/v sucrose and 10% v/v ethanol; and 0.06 kcal/mL is isocaloric to 1.5% w/v sucrose and 1% v/v ethanol). Following habituation, mice were injected with vehicle or CNO (3 mg/kg i.p.) to activate Oxtr^PBN^ neurons (Figure 1b). When fluids were returned after overnight dehydration, we observed that initially mice drank very rapidly (s to min) and then decreased their rate of intake over an extended period (h), so we chose to measure fluid intake at two timepoints: 15 min (initial rapid intake) and 2 h (cumulative intake) (Figure 1c).

In contrast to our previous research, Oxtr^PBN^ neuron activation did decrease 15‐min Ensure® intake at 1 kcal/mL (manufacturer's recommended concentration), but only by ~18% compared to control (vehicle injection) (*F*(1, 13) = 6.81, *p* = 0.02), suggesting a small effect on initial rapid intake (Oxtr^PBN^ activation has previously been observed to decrease non‐caloric fluid intake by ~71% (Ryan et al., 2017)), although the lowest Ensure® drinkers in the vehicle group also tended to be the lowest responders after CNO injection, suggesting that the reduced Ensure® intake might be driven by mice with a low baseline of Ensure® intake (Figure 1d). Similar to previous research, there was no significant difference for 2‐h cumulative intake of 1 kcal/mL Ensure® or water compared to vehicle‐injected mice, suggesting that the CNO‐injected mice had increased their Ensure® intake to the level of control (vehicle‐injected) mice (Ryan et al., 2017) (Figure 1e; Figure S1; Table S2). There was no effect of CNO on Ensure® intake for AAV‐DIO‐tdTomato mice in these studies, nor a difference in water intake (Figure 1d,e; Figure S1; Table S2).

For lower concentrations of Ensure®, Oxtr^PBN^ activation generally reduced both initial 15‐min and cumulative 2‐h intake in a concentration‐related manner (Figure 1d,e; Table S2), demonstrating that activating Oxtr^PBN^ neurons decreased overall intake of lower concentrations of Ensure®. Although we did not formally measure latency to drink, mice were often observed to approach the fluid bottles during the first 15 min and occasionally drink small amounts, before moving to the corner of the cage to rest. Note that there was no significant interaction for 2‐h intake of 0.06 kcal/mL, likely because there were more variable effects on fluid intake suppression at this lower concentration. There was no difference in paired water intake at any of these concentrations of Ensure® (except for the control mice at 0.06 kcal/mL at 15 min, although water intake was very low) (Figure 1d,e; Figure S1; Table S2).

At the end of experimentation, we perfused mice 2 h after CNO injection and examined DREADD reporter and FOS immunoreactivity to validate targeting and activation of Oxtr^PBN^ neurons (Figure 1f,g). As previously noted, we observed viral spread predominantly in the dorsolateral PBN, and some in the external lateral PBN, with occasional minor spread to the adjacent cerebellar region (Figure 1f,g; Figure S2) (Ryan et al., 2017). We observed no significant difference in 15‐min Ensure® intake for unilateral versus bilateral hM_3_Dq injections (*t*(7) = 1.32; *p* = 0.23, unpaired *t*‐test; *n* = 7 bilateral; 2 unilateral), so results were combined (using the average FOS immunoreactivity count for the bilateral PBN injections). We observed an inverse correlation between 15‐min Ensure® (1 kcal/mL) intake and % FOS/Oxtr immunoreactivity in the PBN (*r*(7) = 7.1, *p* = 0.03, Pearson product–moment correlation) (Figure 1h), suggesting that the potency of chemogenetic activation on LPBN neurons (as measured by FOS immunolabelling) predicted their ability to chemogenetically inhibit consumption of Ensure®. The variability in % FOS immunoreactivity may explain why there was a small but significant difference in 1 kcal/mL Ensure® intake at 15 min in the current experiment, but not in previous experiments (Ryan et al., 2017).

### 3.2 Activation of Oxtr^PBN^ neurons suppressed initial rapid intake of all fluids, but not cumulative intake of highly caloric or palatable fluids

**FIGURE 2:**
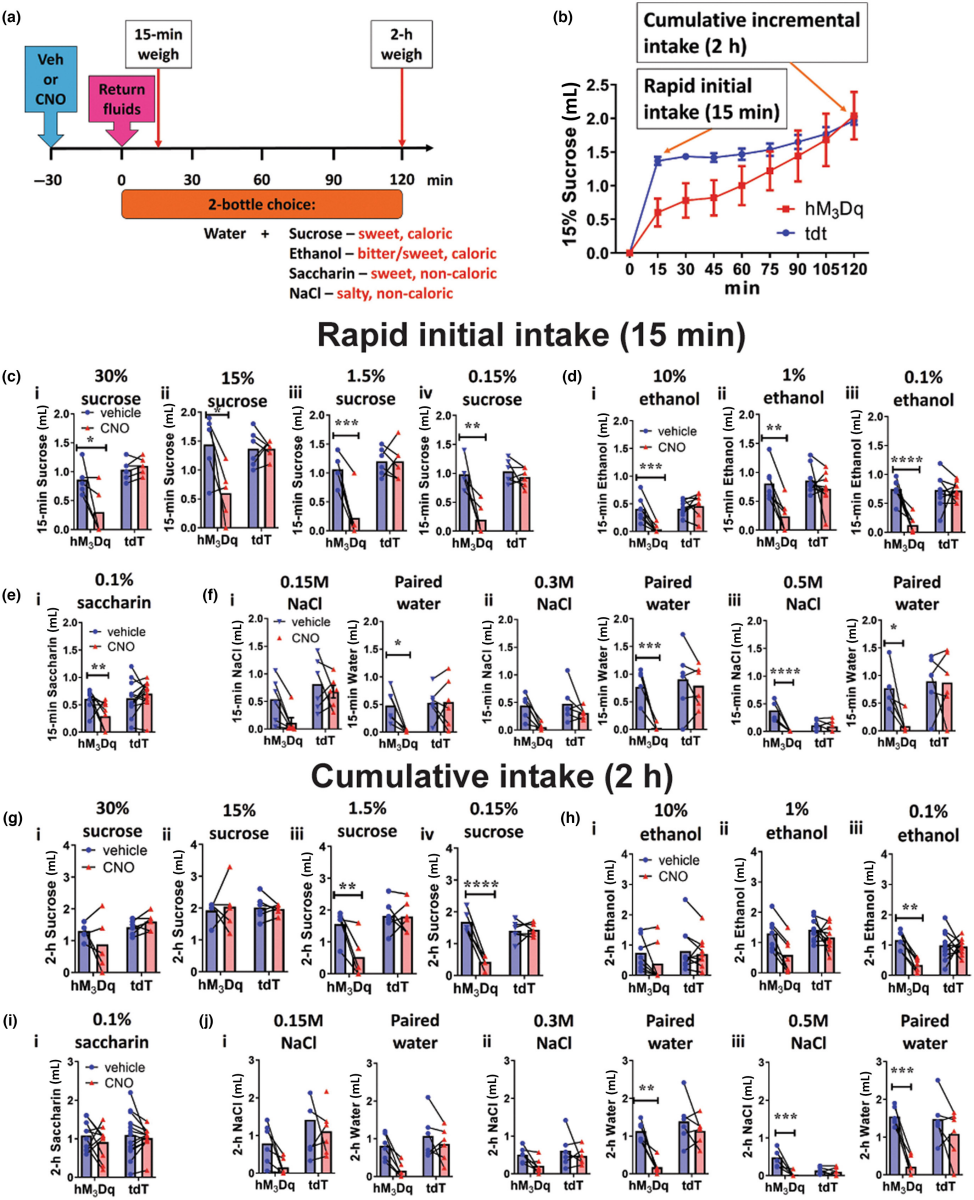
Activation of Oxtr^PBN^ neurons suppressed initial rapid intake of all fluids, but not cumulative intake of highly caloric or palatable fluids. (a) Mice had access for 4 h/day to a two‐bottle choice of water and an experimental solution (sucrose, ethanol, saccharin, saline) at several different concentrations. Mice were injected with vehicle or CNO 3 mg/kg i.p. (b) Intake profile of fluid consumption which includes 15‐min (rapid) and 2‐h (cumulative) intake of fluids. Fluid intake was measured for the initial 15 min of fluid return, which was generally substantial following dehydration; and for cumulative 2‐h intake, when fluid intake had generally plateaued. (c, *i–iv*) Oxtr^PBN^ activation significantly decreased 15‐min rapid intake of sucrose (30%, 15%, 1.5%, 0.15%); paired water intake was not significantly different; *n* = 5 hM_3_Dq, 6 tdTomato. (d, *i–iii*) Oxtr^PBN^ activation significantly decreased 15‐min rapid intake of ethanol (10%, 1%, 0.1%); *n* = 7 hM_3_Dq, 10 tdTomato. (e, *i*) Oxtr^PBN^ activation decreased 15‐min intake of 0.1% w/v saccharin; *n* = 10 hM_3_Dq, 13 tdTomato. (f, *i–iii*) Oxtr^PBN^ activation decreased 15‐min rapid intake of 0.5 M saline. There was no significant interaction for 15‐min intake of 0.15 and 0.3 M NaCl; paired water intake was significantly reduced for 0.15, 0.3 and 0.5 M NaCl; *n* = 6 hM_3_Dq, 6 tdTomato. (g, *i*, *ii*) No significant effect of Oxtr^PBN^ activation was observed for 2‐h cumulative intake of 30% and 15% w/v sucrose. (*iii*, *iv*) Oxtr^PBN^ activation significantly decreased 2‐h intake of lower concentrations of sucrose; *n* = 5 hM_3_Dq, 6 tdTomato. (h, *i*, *ii*) No significant effect of Oxtr^PBN^ activation was observed for 2‐h intake of 10% or 1% ethanol. (*iii*) Oxtr^PBN^ activation significantly decreased 2‐h intake of 0.1% ethanol; *n* = 7 hM_3_Dq, 10 tdTomato. (i, *i*) No significant effect was observed for 2‐h intake of 0.1% w/v saccharin; *n* = 10 hM_3_Dq, 13 tdTomato. (j, *i*) No effect was observed for 2‐h intake of 0.15 M or paired water intake; (*ii*) No effect was observed on 2‐h 0.3 M NaCl, but there was a significant decrease in paired water intake. (*iii*) Oxtr^PBN^ activation significantly decreased 2‐h cumulative intake of 0.5 M NaCl and paired water intake. *n* = 6 hM_3_Dq, 6 tdTomato. Data expressed as mean ± s.e.m.; ****p < 0.0001; ***p < 0.001; **p < 0.01; *p < 0.05.

We investigated the effect of activation of Oxtr^PBN^ neurons on solutions with different caloric and palatability properties, including sucrose, ethanol, saline (NaCl) and saccharin. We injected AAV‐DIO‐hM_3_Dq:mCherry or AAV‐DIO‐tdTomato (control) bilaterally into the PBN of *Oxtr* ^*Cre*^ mice and used a two‐bottle choice paradigm, with water on the one side and the experimental solution on the other (Figure 2a). We examined intake at different concentrations of each solution at 15 min (initial rapid intake) and 2 h (cumulative incremental intake) (Figure 2b).

#### 3.2.1 Sucrose

Oxtr^PBN^ neuron activation significantly decreased initial (15‐min) sucrose intake at all concentrations tested (w/v: 0.15%; 1.5%, 15%, 30%) (Figure 2c; Table S3). Cumulative (2‐h) intake was significantly decreased for lower sucrose concentrations (1.5%, 0.15%), but not higher concentrations (15%, 30%) (Figure 2g; Table S3). There was no difference in paired water intake (except for 2‐h paired water intake for 30% sucrose), although water intake was typically low (Figures S3 and S4; Table S3). Overall, these results suggest that Oxtr^PBN^ neuron activation suppresses initial intake of sucrose at all concentrations, but not 2‐h cumulative intake for higher sucrose concentrations.

#### 3.2.2 Ethanol

Oxtr^PBN^ activation significantly decreased 15‐min intake of ethanol at all concentrations tested (v/v: 0.1%, 1%, 10%) (Figure 2d; Table S3). There was no significant difference in paired water intake, except a significant interaction for paired water associated with 0.1% v/v ethanol (Figure S3; Table S3). For cumulative (2‐h) ethanol intake, we observed significantly decreased intake for 0.1% ethanol, but no significant interaction for 1% or 10% ethanol (Figure 2h; Table S3). There was no significant difference for paired water intake (Figure S4; Table S3). These results demonstrate that the major suppressive effect of Oxtr^PBN^ neuron activation on ethanol also occurs during the initial 15 min.

#### 3.2.3 Saccharin

Oxtr^PBN^ neuron activation decreased 15‐min 0.1% w/v saccharin intake, which has a similar preference value to 15% w/v sucrose but is non‐caloric (Bachmanov et al., 1996; Sclafani et al., 2010) (Figure 2e; Table S3). Paired water intake was not significantly different (Figure 2e; Figure S3; Table S3). At 2 h, there was no significant difference at 0.1% saccharin, nor in paired water intake (Figure 2i; Figure S4; Table S3). These results suggest that Oxtr^PBN^ neurons generally decreased rapid initial drinking, but not cumulative drinking of sweetened, non‐caloric solutions.

#### 3.2.4 Saline (NaCl)

Higher concentrations of NaCl are generally aversive, whereas lower concentrations tend to be more palatable (Bachmanov et al., 2002), so we investigated three concentrations: 0.15 M (isotonic), 0.3 M and 0.5 M. There was no significant interaction for Oxtr^PBN^ activation on 15‐min intake of 0.15 or 0.3 M; however, Oxtr^PBN^ activation decreased initial 15‐min intake of 0.5 M NaCl intake (Figure 2f; Table S3). Paired water intake was significantly reduced at 15‐min for all concentrations, suggesting that the overall effect of Oxtr^PBN^ neuron activation was to decrease paired water intake for a two‐bottle choice of NaCl and water, and decrease 0.5 M NaCl (Figure 2f; Table S3).

There was no significant decrease in 2‐h intake of NaCl, except for 0.5 M NaCl, which is aversive and intake low (Figure 2j; Table S3). Paired water intake remained significantly reduced at 2 h for 0.3 and 0.5 M NaCl (Figure 2j; Table S3). Overall, these results indicate that Oxtr^PBN^ neuron activation decreases the initial paired water intake, and decreases the cumulative paired water intake for 0.3 and 0.5 M NaCl. Given that complementary regulation of NaCl and water maintains body fluid homeostasis (Geerling & Loewy, 2008), it is not surprising that Oxtr^PBN^ neuron activation had more of an effect on the paired water intake in this two‐bottle choice experiment in which NaCl intake was low. At the end of experimentation, we examined DREADD reporter and tdTomato expression to validate the targeting of Oxtr^PBN^ neurons (Figure S5).

Overall, Oxtr^PBN^ neuron activation suppressed rapid, initial (15‐min) rehydration for most solutions by >50%. Cumulative (2‐h) intake remained suppressed for low‐caloric, less palatable solutions, whereas mice increased intake of more highly caloric and/or palatable solutions. This suggests that Oxtr^PBN^ neurons encode a signal which decreases initial homeostatic rehydration responses, but not cumulative intake of highly caloric, palatable solutions.

### 3.3 Activation of Oxtr^PBN^ neurons decreased anxiety‐like behaviour

Activation of Oxtr^PBN^ neurons decreased initial rapid drinking, which we hypothesised may be because of increased anxiety‐like behaviour. Oxtr^PBN^ neurons were chemogenetically activated and mice were tested in elevated plus maze and large open field tests, as well as the locomotor test to assess general movement.

**FIGURE 3:**
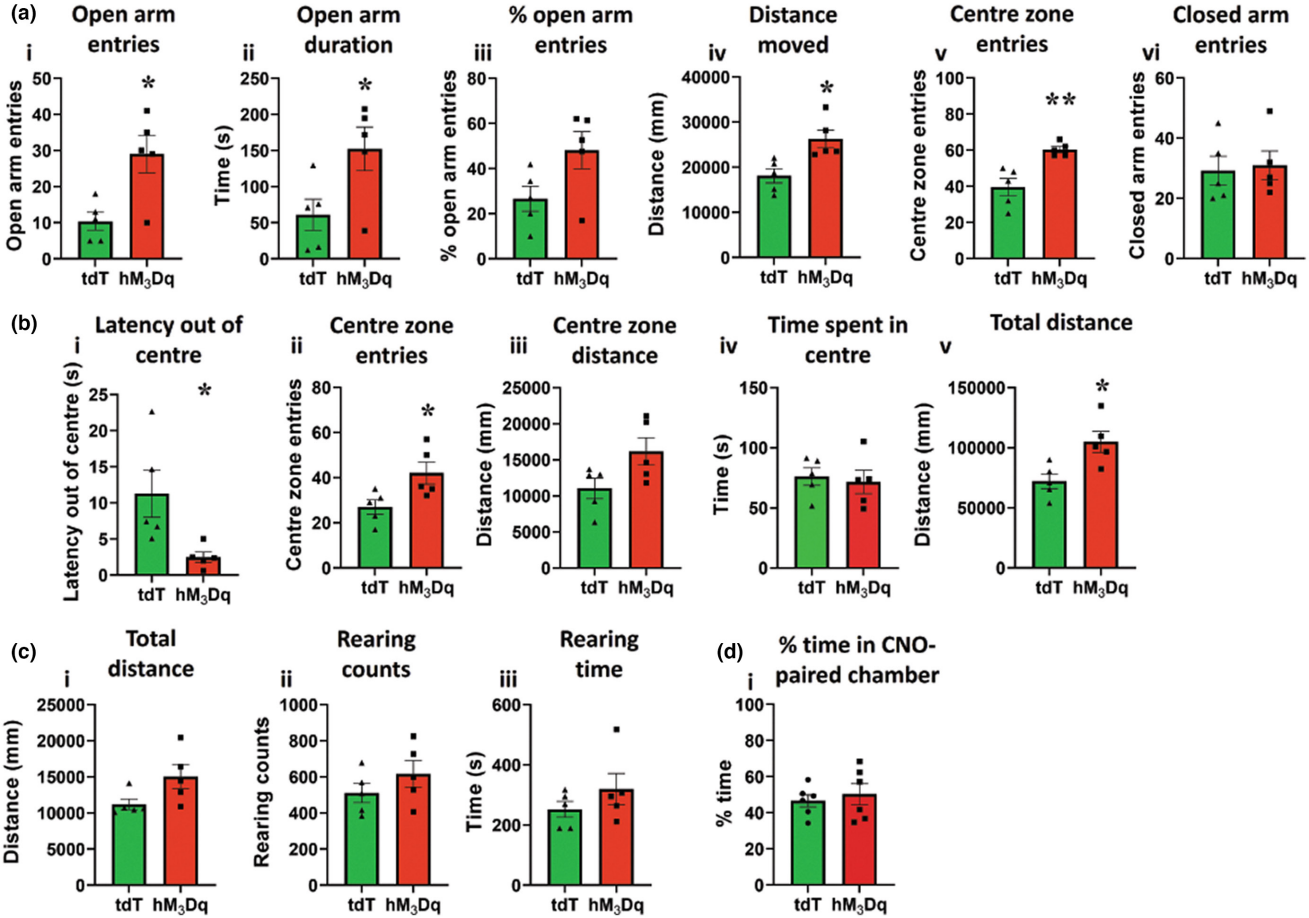
Activation of Oxtr^PBN^ neurons decreased anxiety‐like behaviour. (a) Oxtr^PBN^ neuron activation decreased anxiety‐like behaviour in the elevated plus maze. (*i*) Oxtr^PBN^ neuron activation significantly increased open arm entries; (*ii*) increased duration on open arms; and (*iii*), demonstrated a trend for increased open arm entries as a proportion of total arm entries. (*iv*) Oxtr^PBN^ neuron activation increased total distance moved; (*v*) increased centre zone entries; but (*vi*) no had no change in closed arm entries. Data expressed as mean ± s.e.m.; *n* = 5 hM_3_Dq, 5 tdTomato. *n* = 5 hM_3_Dq, 5 tdTomato. (b) Oxtr^PBN^ neuron activation decreased anxiety‐like behaviour in the large open field. (*i*) Oxtr^PBN^ neuron activation significantly decreased latency to move out of centre; (*ii*) increased entries into centre, and (*iii*) demonstrated a trend for increased centre zone. (*vi*) Oxtr^PBN^ neuron activation did not increase time in centre zone, but (*v*) increased total distance moved; *n* = 5 hM_3_Dq, 5 tdTomato. (c) Oxtr^PBN^ neuron activation did not change distance moved or rearing behaviour in the locomotor cell. (*i*) Oxtr^PBN^ neuron activation did not change total ambulatory distance, or (*ii*) rearing counts, or (*iii*) rearing time; *n* = 5 hM_3_Dq, 5 tdTomato. (d, *i*) Oxtr^PBN^ neuron activation did not change preference for the CNO‐paired chamber in the conditioned place preference paradigm. Data expressed as mean ± s.e.m.; ****p < 0.0001; ***p < 0.001; **p < 0.01; *p < 0.05.

Oxtr^PBN^ neuron activation decreased anxiety‐like behaviour in the elevated plus maze, as observed by increased open arm entries and duration, total distance moved and increased centre zone entries (open arm entries: *t*(8) = 3.2, *p* = 0.01; open arm duration: *t*(8) = 2.46, *p* = 0.04; % open arm entries: *t*(8) = 2.16, *p* = 0.06; total distance moved: *t*(8) = 3.20, *p* = 0.01; centre zone entries: *t*(8) = 4.09, *p* = 0.004; closed arm entries: *t*(8) = 0.27, *p* = 0.80; unpaired *t*‐test) (Figure 3a).

Activation also decreased anxiety‐like behaviour in the large open field, as observed by decreased latency to move out of the centre and increased entries into the centre, as well as increased total distance moved (latency out of centre: *t*(8) = 2.63; *p* = 0.03; centre zone entries: *t*(8) = 2.58; *p* = 0.03; centre zone distance: *t*(8) = 2.18; *p* = 0.06; time in centre zone: *t*(8) = 0.37, *p* = 0.72; total distance moved: *t*(8) = 3.07, *p* = 0.02; unpaired *t*‐test) (Figure 3b), indicating that the fluid‐suppressive effect of Oxtr^PBN^ neuron activation is not because of increased anxiety‐like behaviour. There was no significant difference in total distance moved, rearing counts and rearing times in the locomotor test (total distance: *t*(8) = 2.13; *p* = 0.07; rearing counts: *t*(8) = 1.16; *p* = 0.28; rearing time: *t*(8) = 1.14; *p* = 0.29; unpaired *t*‐test) (Figure 3c), suggesting the effects seen on anxiety‐like behaviour were not due only to locomotor behaviour.

To investigate whether Oxtr^PBN^ neuron activation was pleasurable or aversive, a separate cohort of Oxtr^*Cre*^ mice was tested in the conditioned place preference (CPP) paradigm. After 10 days of conditioning, there was no difference in preference on test (% time in CNO‐paired chamber: *t*(10) = 0.53; *p* = 0.61) (Figure 3d), suggesting that activation of Oxtr^PBN^ neurons was neither pleasurable nor aversive.

### 3.4 Different solutions activated different LPBN subdivisions depending on fluid, caloric content and palatability

**FIGURE 4:**
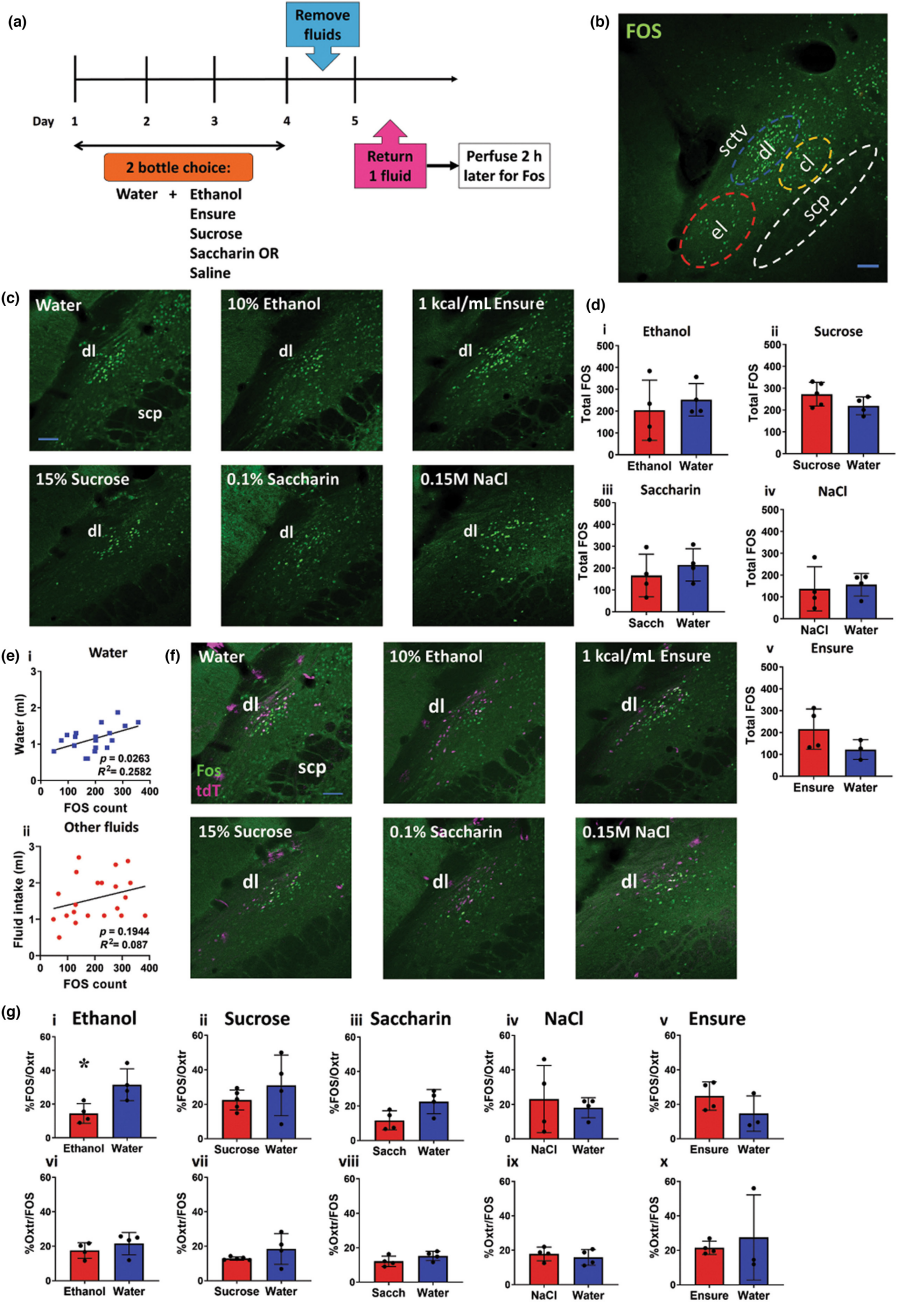
Different solutions increased FOS immunoreactivity in the dl LPBN. (a) Oxtr^Cre^::Ai14 mice had access for 4 h/day to a two‐bottle choice of water and an experimental solution (10% v/v ethanol; Ensure® 1 kcal/mL; 15% w/v sucrose; 0.1% saccharin; 0.15 M NaCl). Fluid was removed for 24 h, then either water or experimental fluid returned 2‐h prior to perfusion. (b) Reference image of the LPBN demonstrating FOS immunoreactivity in different subsections following 1 kcal/mL Ensure® intake: dl = dorsolateral; el = external lateral; cl = central lateral; scp = superior cerebellar peduncle; sctv = ventral spinocerebellar tract. Scale bar, 100 μm. Bregma −5.3 mm. (c) FOS immunoreactivity in the dl LPBN following fluid intake after 24‐h dehydration. Representative images of neural activation in the dl LPBN following consumption of all fluids tested. *n* = 3–5 Oxtr^Cre^::Ai14 group. Scale bar, 100 μm; scp = superior cerebellar peduncle. Bregma −5.3 mm. (d) FOS immunoreactivity in the dl LPBN following fluid intake. (*i–v*) No significant difference was observed in FOS immunoreactivity between experimental fluid compared to water consumption. (e, *i*) A significant correlation between 30‐min water intake and FOS‐positive cells counted was observed in the dl LPBN. (*ii*) No significant correlation was observed between 30‐min intake of other fluids and FOS immunoreactivity; *n* = 3–5/group Oxtr^Cre^::Ai14 reporter mice. (f) FOS‐Oxtr colocalisation in the dl LPBN following fluid intake. Representative images of colocalisation of Oxtr with FOS in the dl LPBN for all fluids tested. Scale bar, 100 μm; scp = superior cerebellar peduncle. Bregma −5.3 mm. (g) % FOS/Oxtr and % Oxtr/FOS colocalisation in the dl LPBN. (*i*) There was a significant decrease in % FOS in Oxtr^PBN^ neurons for ethanol compared to water. (*ii–v*) No significant difference in % FOS/Oxtr for 15% w/v sucrose, 0.1% w/v saccharin, 0.15 M NaCl or 1 kcal/mL Ensure® was identified. (*vi–x*) No significant difference in % Oxtr/FOS immunoreactivity was observed; *n* = 40 Oxtr^Cre^::Ai14 reporter mice.

We hypothesised that the differences in fluid intake after Oxtr^PBN^ activation for different solutions might be because of differences in neural activation in the LPBN, particularly the fluid‐related dl LPBN, so we examined FOS immunoreactivity in the LPBN following 1 kcal/mL Ensure®; 10% ethanol; 15% sucrose; 0.1% saccharin; and 0.15 M saline. In each experiment, *Oxtr* ^*Cre*^::Ai14 mice were habituated to a two‐bottle choice paradigm of water and an experimental solution (Ensure®, ethanol, sucrose, saccharin or saline), then dehydrated for 24 h and returned either water or the experimental solution 2 h prior to perfusion (Figure 4a). We observed FOS immunoreactivity primarily in dorsolateral (dl), external lateral (el) and central lateral (cl) subdivisions of the LPBN (Figure 4b).

After water intake, we observed increased FOS predominantly in the dl LPBN, as described (Ryan et al., 2017). Surprisingly, there were no significant differences in FOS immunoreactivity in the dl LPBN for any experimental fluid (ethanol, sucrose, saccharin, Ensure® or saline) compared to water intake, suggesting that differences in fluid intake do not arise from overall differences in dl LPBN activation (10% v/v ethanol: *t*(6) = 0.61, *p* = 0.56; 15% w/v sucrose: *t*(7) = 1.63, *p* = 0.15; 0.1% w/v saccharin: *t*(6) = 0.80, *p* = 0.46; 0.15 M NaCl: *t*(6) = 0.33, *p* = 0.75; 1 kcal/mL Ensure®: *t*(5) = 1.59; *p* = 0.17; unpaired *t*‐test) (Figure 4c,d). We observed a correlation of FOS immunoreactivity with water intake in the dl LPBN (*r*(17) = 0.51, *p* = 0.03; Pearson product–moment correlation), but not other fluids (*r*(19) = 0.30, *p* = 0.19; Pearson product–moment correlation) (Figure 4e).

We also examined FOS immunoreactivity specifically in Oxtr‐expressing neurons of the dl LPBN, and observed a similar percentage FOS immunoreactivity in all fluids (For % FOS/Oxtr: 10% v/v ethanol: *t*(6) = 3.06, *p* = 0.02; 15% w/v sucrose: *t*(7) = 1.03, *p* = 0.34; 0.15% w/v saccharin: *t*(6) = 2.44, *p* = 0.05; 0.15 M NaCl: *t*(6) = 0.50, *p* = 0.64; 1 kcal/mL Ensure®: *t*(5) = 1.47, *p* = 0.20. For % Oxtr/FOS: 10% v/v ethanol: 10% v/v ethanol: *t*(6) = 1.01, *p* = 0.35; 15% w/v sucrose: *t*(7) = 1.41, *p* = 0.20; 0.1% w/v saccharin: *t*(6) = 1.52, *p* = 0.18; 0.15 M NaCl: *t*(6) = 0.65, *p* = 0.54; 1 kcal/mL Ensure®: *t*(5) = 0.49, *p* = 0.64; unpaired *t*‐test) (Figure 4f,g). Note that there was a lower % FOS/Oxtr immunoreactivity in ethanol versus water, although absolute amounts of FOS were similar, suggesting that some FOS was expressed in non‐Oxtr‐expressing neurons (Figure 4f,g).

**FIGURE 5:**
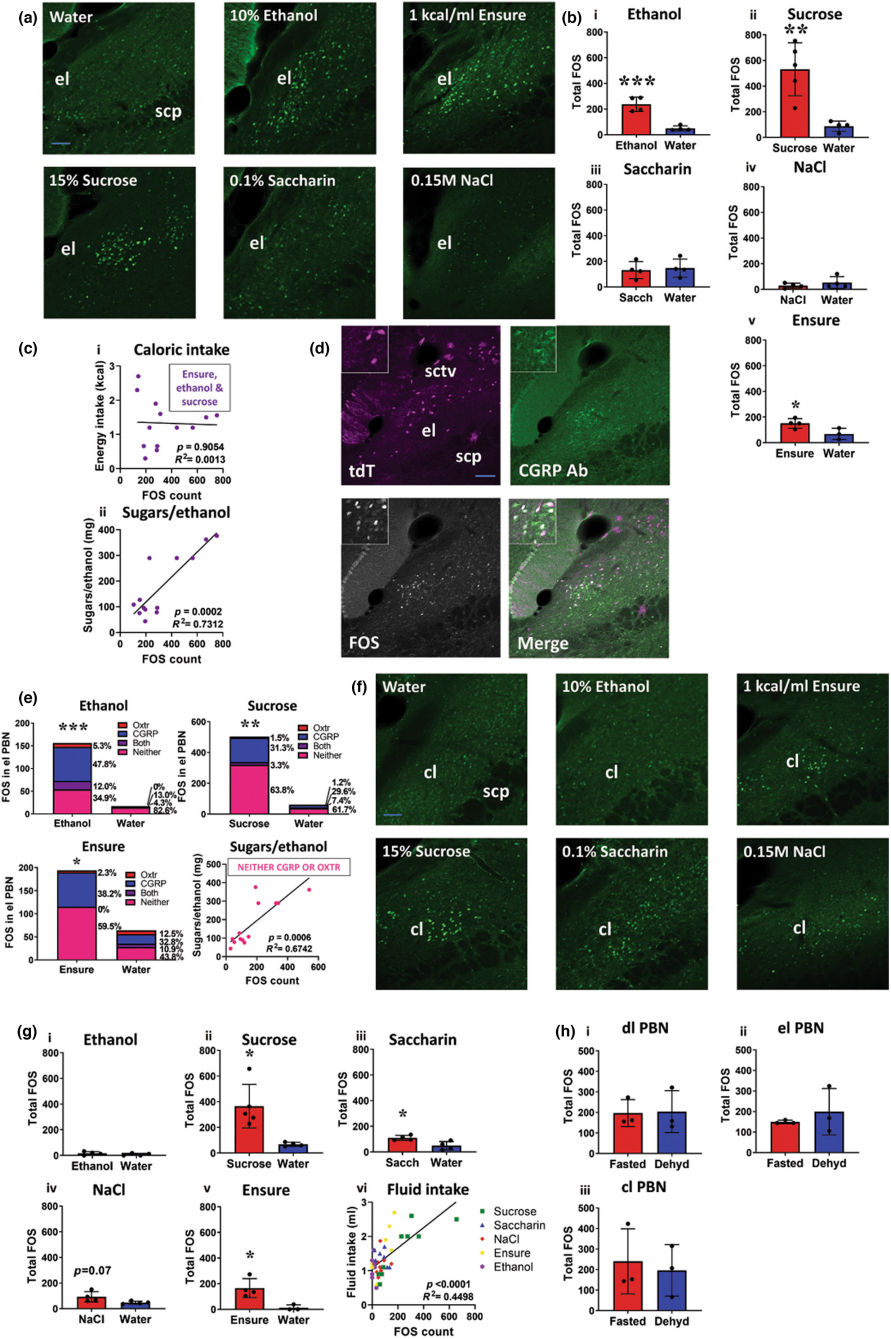
Different solutions increased FOS immunoreactivity in the el and cl LPBN. (a) FOS immunoreactivity in the el LPBN following fluid intake after 24‐h dehydration. Representative images of neural activation in the el LPBN following consumption of caloric fluids. Scale bar, 100 μm. *n* = 3–5 Oxtr^Cre^::Ai14 group. Bregma −5.2 mm. (b) Total FOS immunoreactivity in the el LPBN following fluid intake. (*i*, *ii*) A significant increase in total FOS expressed in the elPBN was observed for 10% v/v ethanol and 15% w/v sucrose. (*iii*, *iv*) No significant difference in total FOS immunoreactivity was observed for 0.1% w/v saccharin or 0.15 M NaCl. (*v*) A significant increase in FOS immunoreactivity in the el LPBN was observed for Ensure®. (c, *i*) No significant correlation was observed between el LPBN FOS immunoreactivity and caloric energy intake. (*ii*) A significant correlation was observed between sugar/ethanol intake (mg) and FOS immunoreactivity in the el LPBN; *n* = 40 Oxtr^Cre^::Ai14 reporter mice for total FOS analysis; *n* = 13 Oxtr^Cre^::Ai14 reporter mice for correlation analysis. (d) FOS‐Oxtr‐CGRP colocalisation in the el LPBN following fluid intake. Representative image for colocalisation of Oxtr (tdT) and CGRP with FOS in the el LPBN following caloric fluid intake. Scale bar, 100 μm. Bregma −5.2 mm. (e) Colocalisation of FOS‐Oxtr‐CGRP following caloric fluid intake. A significant increase in total FOS expressed in the el LPBN was observed for 10% v/v ethanol, 15% w/v sucrose and 1 kcal/mL Ensure® compared to water, and there was a significant correlation between sugar/ethanol (mg) intake and Fos‐expressing non‐CGRP/Oxtr neurons were observed (non‐CGRP/Oxtr: *r*(11) = 0.821, *p* = 0.0006); Pearson product–moment correlation; *n* = 27 *Oxtr* ^*Cre*^::Ai14 reporter mice. (f) Total FOS immunoreactivity in the cl LPBN following fluid intake after 24‐h dehydration. Representative images of neural activation in the cl LPBN following consumption of highly palatable and sweet tasting fluids. Scale bar, 100 μm. *n* = 3–5 Oxtr^Cre^::Ai14 group. Scale bar, 100 μm. Bregma −5.3 mm. (g) Total FOS immunoreactivity in the cl LPBN following fluid intake. (*i*) No significant difference was observed in total FOS immunoreactivity in the cl LPBN following 10% v/v ethanol intake compared to water. (*ii*, *iii*) A significant increase in total FOS expressed in the cl LPBN was identified for 15% w/v sucrose and 0.1% w/v saccharin. (*iv*) No significant difference in total FOS immunoreactivity in the cl LPBN following 0.15 M NaCl intake was observed; however, there was a trend towards significance. (*v*) Total FOS immunoreactivity in the cl LPBN following 1 kcal/mL Ensure® intake was significantly elevated compared to water. (*vi*) A significant correlation was observed between 30‐min fluid intake and FOS‐positive cells counted in the cl LPBN; *n* = 40 Oxtr^Cre^::Ai14 reporter mice. Data expressed as mean ± s.e.m.; ****p < 0.0001; ***p < 0.001; **p < 0.01; *p < 0.05. (h) Total FOS immunoreactivity in PBN subdivisions following Ensure® intake after fasting versus dehydration. (*i*–*iii*) No significant change in total FOS immunoreactivity was observed in any PBN subdivision following the return of 1 kcal/mL Ensure® after fasting or dehydration; *n* = 3 fasted, 3 dehydrated Oxtr^Cre^::Ai14 reporter mice. scp, superior cerebellar peduncle; sctv, ventral spinocerebellar tract.

In the el LPBN, we observed increased FOS immunoreactivity primarily after ethanol, sucrose and Ensure® intake, but not saccharin, saline or water (10% v/v ethanol: *t*(6) = 6.30, *p* = 0.0007; 15% w/v sucrose: *t*(7) = 4.20, *p* = 0.004; 0.1% w/v saccharin: *t*(6) = 0.32, *p* = 0.76; 0.15 M NaCl: *t*(6) = 0.97, *p* = 0.37; 1 kcal/mL Ensure®: *t*(5) = 2.70, *p* = 0.04; unpaired *t*‐test) (Figure 5a,b). FOS immunoreactivity in the el LPBN was correlated with sucrose/ethanol intake but not calories ingested (sugar/ethanol (mg): *r*(11) = 0.86, *p* = 0.0002; caloric intake: *r*(11) = −0.04, *p* = 0.91; Pearson product–moment correlation) (Figure 5c).

In the el LPBN, we examined FOS immunoreactivity in both Oxtr‐ and CGRP‐expressing neurons following intake of caloric fluids (Ensure®, ethanol and sucrose). There was a significant increase in total FOS in the el LPBN following intake of caloric fluids compared to water (10% v/v ethanol: *t*(8) = 8.73, *p* < 0.0001; 15% w/v sucrose: *t*(7) 3.75, *p* = 0.007; 1 kcal/mL Ensure®: *t*(6) = 4.32, *p* = 0.005; unpaired *t*‐test), with 31%–48% in CGRP‐expressing neurons, 1.5%–5.3% in Oxtr‐expressing neurons and 0%–12% in neurons that expressed both Oxtr and CGRP. There was also a large population of FOS‐expressing neurons (35%–64%) that were neither CGRP‐ nor Oxtr‐expressing, suggesting an unknown population(s) of el LPBN neurons that respond to caloric fluid intake (Figure 5d,e).

We also observed increased FOS immunoreactivity in the cl LPBN primarily following intake of sucrose, saccharin and Ensure® (which are highly palatable), and there was a trend for an increase in FOS immunoreactivity in the cl LPBN for saline (10% v/v ethanol: *t*(6) = 1.08, *p* = 0.32; 15% w/v sucrose: *t*(7) = 3.45, *p* = 0.01; 0.1% w/v saccharin: *t*(6) = 3.21, *p* = 0.02; 0.15 M NaCl: *t*(6) = 2.17, *p* = 0.07; 1 kcal/mL Ensure®: *t*(5) = 3.39, *p* = 0.02; unpaired *t*‐test) (Figure 5f,g). The cl LPBN is known to contain neurons sensitive to sweet and salt taste (Tokita & Boughter, 2016; Yamamoto et al., 1994). There was a correlation between fluid intake and FOS‐positive cells in the cl LPBN (*r*(38) = 0.67, *p* < 0.0001; Pearson product–moment correlation) (Figure 5g).

We also compared FOS immunoreactivity in the LPBN following 24‐h fasting versus 24‐h dehydration, and observed similar immunoreactivity levels, suggesting that the underlying state does not alter neuronal activation in the LPBN (dl PBN: *t*(4) = 0.10, *p* = 0.93; el PBN: *t*(4) = 0.77, *p* = 0.49; cl PBN: *t*(4) = 0.38, *p* = 0.72) (Figure 5h).

These results suggest that the difference in fluid intake is not because of overall differences in dl LPBN activity because this subdivision is activated similarly by ingestion of fluids regardless of their caloric content or palatability. By contrast, the el LPBN is activated following ingestion of caloric substances (ethanol, sucrose, Ensure®), and the cl PBN is primarily activated following ingestion of sweet‐tasting or highly palatable solutions (sucrose, saccharin, Ensure®).

### 3.5 Activation of CGRP^PBN^ neurons suppressed Ensure® intake at all concentrations

Given that CGRP^PBN^ neurons demonstrated increased FOS immunoreactivity following 1 kcal/mL Ensure® intake, but not lower‐caloric solutions, we hypothesised that activation of CGRP^PBN^ neurons might decrease higher‐caloric Ensure® intake more than lower caloric Ensure®. We injected AAV‐DIO‐hM_3_Dq:mCherry bilaterally into the PBN of *Calca* ^*Cre*^ mice (the *Calca* gene encodes CGRP), or AAV‐DIO‐tdTomato as a control. We gave mice access to a two‐bottle choice of water and Ensure® at different concentrations for 4 h/day. Following habituation, mice were injected with CNO (3 mg/kg i.p.) to activate CGRP^PBN^ neurons.

**FIGURE 6:**
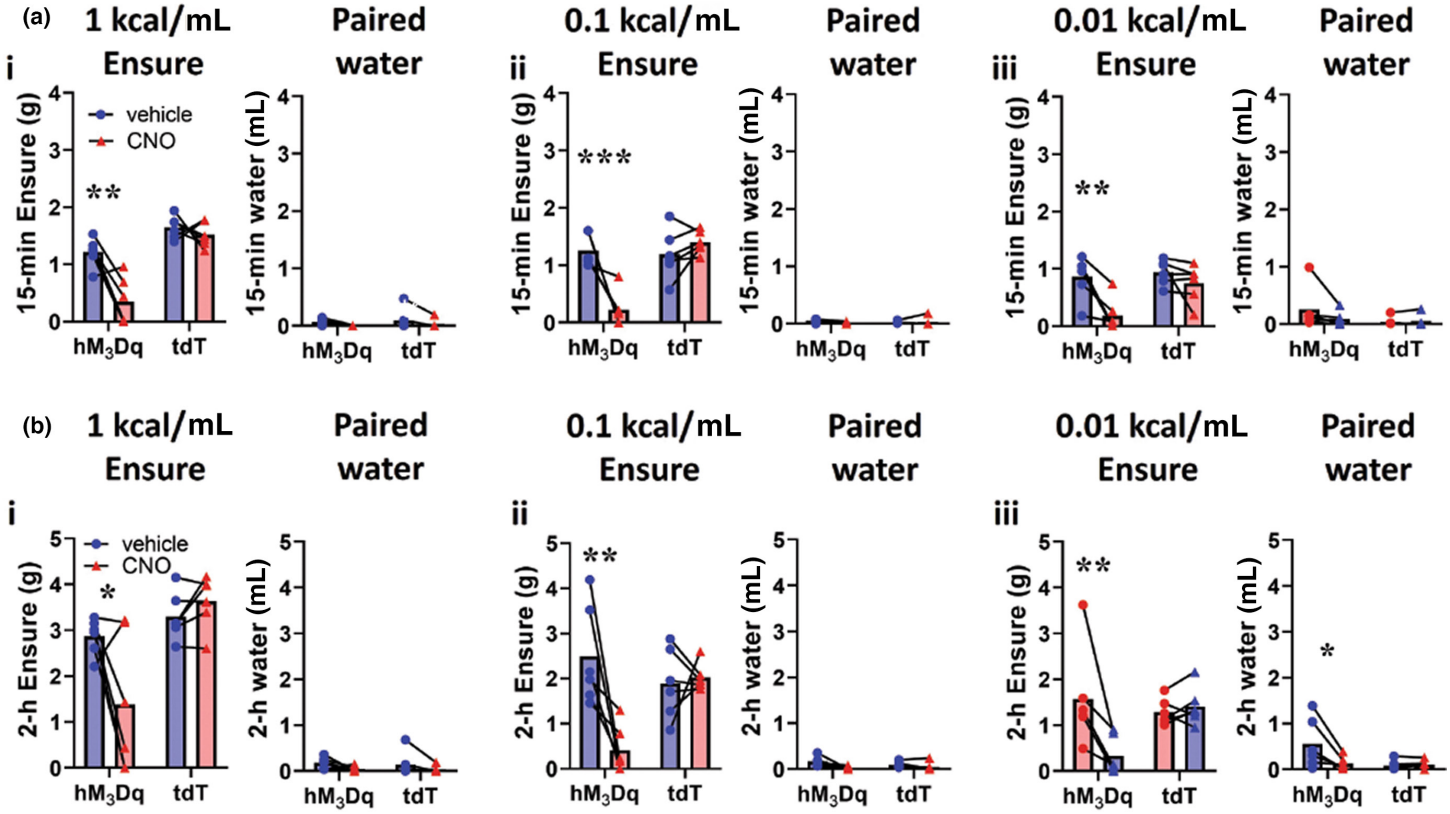
Activation of CGRP^PBN^ neurons suppressed Ensure® intake at all concentrations. (a) CGRP^PBN^ activation decreased 15‐min rapid intake of Ensure®. Acute CGRP^PBN^ stimulation significantly decreased rapid 15‐min intake of (*i*) 1 kcal/mL Ensure®; (*ii*) 0.1 kcal/mL Ensure®; (*iii*) 0.01 kcal/mL Ensure®, but did not significantly decrease paired water intake; *n* = 6 hM_3_Dq, 6 tdTomato. (b) CGRP^PBN^ activation decreased 2‐h rapid intake of Ensure®. Acute CGRP^PBN^ stimulation significantly decreased rapid 2‐h intake of (*i*) 1 kcal/mL Ensure®; (*ii*) 0.1 kcal/mL Ensure®; (*iii*) 0.01 kcal/mL Ensure®, but did not significantly decrease paired water intake, except at 0.01 kcal/mL Ensure®; however, water intake was low; *n* = 6 hM_3_Dq, 6 tdTomato. Data expressed as mean ± s.e.m.; ****p < 0.0001; ***p < 0.001; **p < 0.01; *p < 0.05.

We observed a significant decrease in Ensure® intake at all concentrations at 15‐min and 2‐h time points, suggesting that activation of CGRP^PBN^ neurons suppresses both caloric and non‐caloric intake similarly (15‐min 1 kcal/mL Ensure®: *F*(1, 1) = 6.90, *p* = 0.03; 15‐min 0.1 kcal/mL Ensure®: *F*(1, 10) = 24.19, *p* = 0.0006; 15‐min 0.01 kcal/mL Ensure®: *F*(1, 10) = 5.35, *p* = 0.04; 2‐h 1 kcal/mL Ensure®: *F*(1, 10) = 7.12, *p* = 0.02; 2‐h 0.1 kcal/mL Ensure®: *F*(1, 10) = 10.05, *p* = 0.01; 2‐h 0.01 kcal/mL Ensure®: *F*(1, 10) = 13.74, *p* = 0.004; 2‐way RM ANOVA) (Figure 6a,b).

There was no significant difference for paired water intake (except for 15‐min water intake paired with 0.1 kcal/mL Ensure® and 2‐h water intake paired with 0.01 kcal/mL Ensure®); however, water intake was low in both cases (15‐min paired water for 1 kcal/mL Ensure®: *F*(1, 10) = 0.00, *p* > 0.9999; 15‐min paired water for 0.1 kcal/mL Ensure®: *F*(1, 10) = 5.23, *p* = 0.045; 15‐min paired water for 0.01 kcal/mL Ensure®: *F*(1, 10) = 2.75, *p* = 0.12; 2‐h paired water for 1 kcal/mL Ensure®: *F*(1, 10) = 0.10, *p* = 0.76; 2‐h paired water for 0.1 kcal/mL Ensure®: *F*(1, 10) = 3.78, *p* = 0.08; 2‐h paired water for 0.01 kcal/mL Ensure®: *F*(1, 10) = 7.07, *p* = 0.02; unpaired *t*‐test) (Figure 6a,b).

### 3.6 AgRP inhibited Oxtr^PBN^ neuron activity

We next investigated a potential mechanism to explain why Ensure® intake was not suppressed to the same extent as water or other solutions following activation of Oxtr^PBN^ neurons, given two conflicting results: (1) Oxtr^PBN^ neuron activity does not increase when mice start drinking Ensure® (unlike water) based on in vivo calcium imaging data (Ryan et al., 2017); however, (2) Oxtr^PBN^ neurons demonstrate increased neuronal activation (as evidenced by increased FOS immunoreactivity) after ingesting Ensure® and water (see Figure 4c,d). This suggests that Oxtr^PBN^ neuron activity may be inhibited initially when drinking Ensure®, but then activated in a delayed manner so we hypothesised that there might be an initial inhibitory input to Oxtr^PBN^ neurons that is engaged when mice are exposed to and commence drinking Ensure®.

**FIGURE 7:**
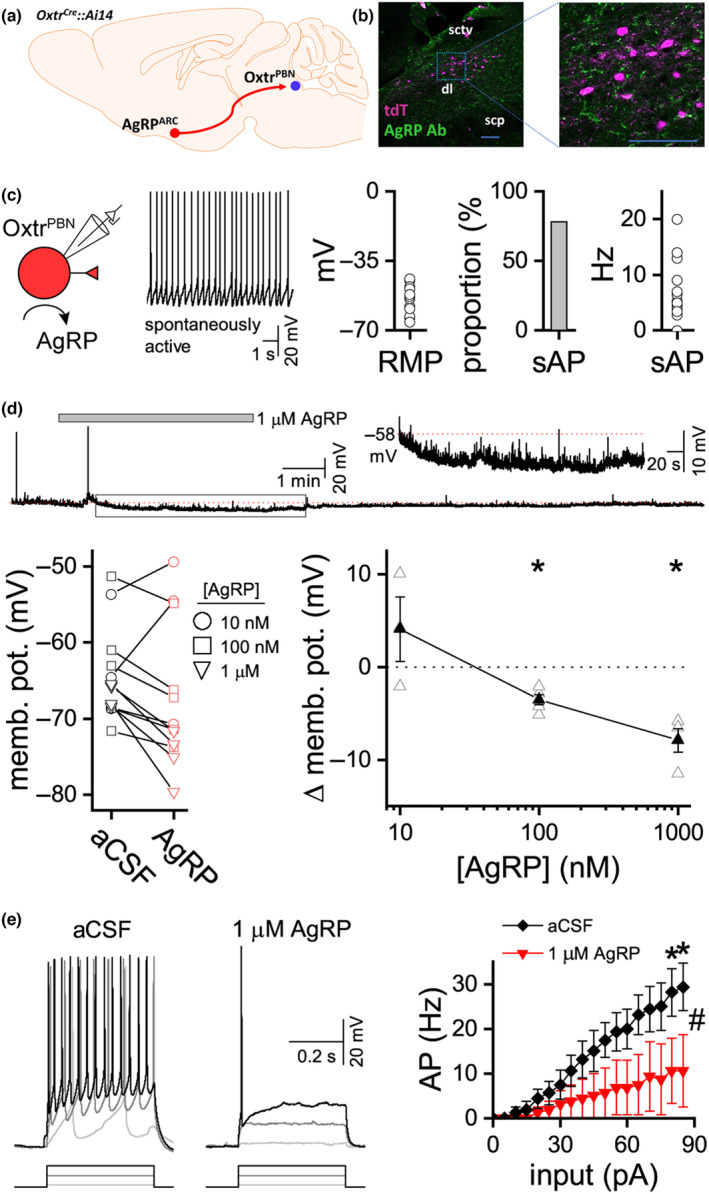
AgRP hyperpolarised Oxtr^PBN^ neurons in a dose‐related manner. (a) Schematic of AgRP^ARC^ neurons projecting to Oxtr^PBN^ neurons in *Oxtr* ^*Cre*^::Ai14 mice. (b) Multiple AgRP fibres adjacent to and surrounding Oxtr^PBN^ neurons (labelled tdT); *n* = 3 *Oxtr* ^*Cre*^::Ai14 mice; scale bar 100 μm; scp = superior cerebellar peduncle; sctv = ventral spinocerebellar tract. (c) Most Oxtr^PBN^ neurons exhibit spontaneous spiking at resting membrane potential (RMP, *n* = 18). (d), AgRP hyperpolarised Oxtr^PBN^ neurons in a dose‐related manner (10 nM, *n* = 3; 100 nM, *n* = 5; and 1 μM, *n* = 4). (e) In the presence of AgRP, greater current input was needed to achieve the same action potential output (*n* = 4). Individual neurons represented as open symbols, mean ± s.e.m. as filled symbols; *p < 0.05, ^#^ *p* < 0.05 for interaction of AgRP and current input.

We hypothesised that this neural input might arise from hunger‐responsive AgRP neurons in the arcuate nucleus (AgRP^ARC^) which send inhibitory projections to the LPBN (Campos et al., 2016) and respond to caloric solutions in a concentration‐related manner (Beutler et al., 2017; Su et al., 2017) (Figure 7a). Previous studies have demonstrated that stimulating AgRP^ARC^ → PBN projections does not increase food intake at baseline (Atasoy et al., 2012), but does increase food intake following injection of appetite‐suppressive compounds such as amylin, cholecystokinin and lithium chloride (Essner et al., 2017), suggesting that stimulating AgRP^ARC^ → PBN projections may inhibit appetite‐suppressive signals, such as satiation. AgRP neurons are known to release AgRP, NPY and GABA from their terminals (Chen et al., 2019; Krashes et al., 2013). Despite AgRP neurons themselves being inhibited often before feeding begins (Chen et al., 2015), the co‐released neuropeptides can generate longer‐lasting responses (Krashes et al., 2013), so we investigated the possible effect of AgRP on Oxtr^PBN^ neurons.

We observed AgRP projections in the LPBN using immunohistochemistry, with relatively sparse branches in the external lateral and dorsolateral LPBN, and with some abutting Oxtr^PBN^ neurons, as anticipated from previous studies (Gasparini et al., 2019) (Figure 7b). Given that AgRP‐expressing neurons are co‐mingled with Oxtr‐expressing neurons in the arcuate nucleus (Fenselau et al., 2017), we did not crossbreed *AgRP* ^*Cre*^ with *Oxtr* ^*Cre*^ mice. Instead, we recorded from Oxtr^PBN^ neurons in slices after bath application of AgRP.

AgRP is selective for MC3R and MC4R (McNulty et al., 2001), where it acts as an inverse agonist and antagonist (Haskell‐Luevano et al., 2001; Ollmann et al., 1997). AgRP can counteract the endogenous melanocortin receptor agonist, α‐MSH, at 10 nM (Cowley et al., 1999) and suppress constitutive activity in MC3R and MC4R‐expressing cells at 100 nM (Nijenhuis et al., 2001), so we used these concentrations for this experiment. Single‐cell sequencing of the LPBN has recently revealed MC4R expression throughout the dorsolateral and external lateral subdivisions of the LPBN (Pauli et al., 2022).

To determine if Oxtr^PBN^ neurons were responsive to AgRP, we made whole‐cell recordings from these neurons from *Oxtr* ^*Cre*^::Ai14 mice and exposed them to 10, 100 or 1000 nM AgRP (Cowley et al., 1999; Fu & van den Pol, 2008; Nijenhuis et al., 2001) (Figure 7c). Oxtr^PBN^ neurons had a resting membrane potential of −53. 5 ± 1.5 mV, where 78% of the cells exhibited spontaneous spiking at a rate of 7.5 ± 1.3 Hz (*n* = 18; Figure 6a). To test for responsiveness to AgRP, neurons were hyperpolarised (−25.5 ± 5.7 pA) for accurate input‐frequency assessment before exposure. In all Oxtr^PBN^ neurons tested (*n* = 12), AgRP exposure at 100 and 1000 nM significantly decreased membrane potential by 3.6 ± 0.5 and 7.9 ± 1.3 mV respectively (Figure 7d). Consequently, corresponding input‐frequency curves were shifted rightward in the presence of AgRP (Figure 7e). This suggests that AgRP may provide inhibitory input to Oxtr^PBN^ neurons and may contribute to the concentration‐related inhibition of fluid satiation for calorie‐rich solutions, although further research is required to confirm these effects in vivo.

## 4 DISCUSSION

These results demonstrate that activation of Oxtr^PBN^ neurons acts to decrease rapid initial fluid intake after dehydration. For most fluids (water, sucrose, ethanol, saccharin), Oxtr^PBN^ activation suppressed initial 15‐min rapid intake by >50%; however, Ensure® (1 kcal/mL) was only decreased by ~18%. In chemogenetic studies, we observed that % FOS immunoreactivity in Oxtr^PBN^ neurons inversely correlated with 15‐min Ensure® intake (see Figure 1h), suggesting mice with increased neuronal activation in the LPBN decreased Ensure® more robustly. It is possible that mice were nauseated, although this is less likely given that they exhibited decreased anxiety‐like behaviour, which would not be expected if mice were unwell. In addition, we observed occasional unintended spread into the cerebellum (5 of 32 hM_3_Dq‐injected mice); however, because of its inconsistent nature, and the typically small number of transduced neurons, it is unlikely that this would meaningfully contribute to the fluid intake behaviour.

For low‐caloric and less palatable solutions (water and saline), fluid intake remained low over 2 h; whereas fluid intake increased to control levels for highly caloric substances (Ensure®, ethanol, sucrose) or sweetened non‐caloric solutions, such as saccharin. This suggests that there are two distinct aspects involved in drinking behaviour: one which controls the initial, rapid drinking of all fluids after dehydration, which can be suppressed by Oxtr^PBN^ activation; and another which controls incremental drinking over 2 h, which favours highly palatable, caloric solutions in a concentration‐related manner. It is also possible that CNO only produces a transient effect on fluid intake; however, given that we have previously demonstrated that Oxtr^PBN^ neurons remain active even 2 h after CNO injection, with an ongoing decrease in water intake that lasts >4 h (Ryan et al., 2017), it is more likely that CNO produces a longer‐lasting effect. We also note that it was unusual that the hM_3_Dq‐injected mice drank some 0.5 M NaCl under control conditions. It is possible that expressing hM_3_Dq may alter the baseline function of some Oxtr^PBN^ neurons in a way that reduces aversion to 0.5 M NaCl, although a previous study did not demonstrate differences in aversive salt concentrations (0.3 or 0.5 M) when hM_3_Dq was injected into Oxtr^PBN^ neurons compared to control viral injections (Ryan et al., 2017) suggesting this is unlikely.

Under physiological conditions, there were no significant differences in FOS immunoreactivity in the dl LPBN (fluid satiation subdivision) after intake of many different solutions. These experiments were timed such that FOS immunoreactivity denoted neural activation at approximately 30 min after fluid intake, so our results suggest that differences in intake of different solutions were not because of differences in overall neural activity in the dl LPBN (see Figure 4c,d). Instead, we hypothesised that an inhibitory input to Oxtr^PBN^ neurons is engaged when mice commence drinking Ensure®. In support of this, we observed that AgRP robustly inhibited Oxtr^PBN^ neurons in a concentration‐related manner, indicating that Oxtr^PBN^ neurons likely express melanocortin receptors and receive an AgRP neuron input to inhibit activity in fluid satiation‐related neurons (Oxtr^PBN^) (see Figure 7). Most Oxtr^PBN^ neurons were spontaneously active, so the consequences of AgRP hyperpolarising these neurons would presumably be first to reduce firing frequency, and second to decrease the impact of excitatory input. This possibly explains the increased intake of higher‐caloric Ensure® intake observed after Oxtr^PBN^ neuron activation. Studies suggest that AgRP may increase feeding over the medium‐ to long‐term (hours) (Krashes et al., 2013), which aligns with our results showing that high‐caloric Ensure® intake continues despite CNO stimulation of Oxtr^PBN^ neurons (Ryan et al., 2017).

Although there is no direct electrophysiological evidence that AgRP neurons synapse directly onto Oxtr^PBN^ neurons, we have immunohistochemical evidence that they are adjacent to and surround Oxtr^PBN^ neurons, but we note that this connectivity does not necessitate a monosynaptic connection (see Figure 7b). Notably, while fast neurotransmitters like glutamate require monosynaptic connections, neuropeptide release is not restricted to synaptic specialisation, and after release, a neuropeptide may diffuse some distance to exert its action (Van Den Pol, 2012), so it is possible that AgRP may exert its effects either monosynaptically or via diffusion. However, further investigation would be useful to identify the connectivity of AgRP neurons and different PBN subsets. The single‐cell sequencing of the PBN revealed Mc4r in the dorsolateral and external lateral subdivisions of the PBN (Pauli et al., 2022), although they did not formally investigate for colocalisation of Mc4r and Oxtr.

AgRP is selective for Mc3R and Mc4R (McNulty et al., 2001), so its effect on Oxtr^PBN^ neurons is functional evidence that these neurons express either Mc3R or Mc4R. Based on single‐cell sequencing data, Mc4R expression is observed in the dl and el LPBN (which is where we recorded Oxtr^PBN^ neurons), so is the likely target of the AgRP; however, there may be Mc3R expression within the PBN, although in a different subregion, so it is another possible target (Pauli et al., 2022).

We observed increased intake of highly palatable, caloric fluids compared to less palatable solutions over the 2‐h period (termed ‘cumulative intake’). This may also be because of AgRP inhibition of Oxtr^PBN^ signalling, although this would not explain the increase in non‐caloric saccharin intake over the 2‐h period. Alternatively, or additionally, it is possible that reward‐related neural circuits may be engaged by highly caloric, palatable solutions, which increases their intake. Consistent with this, a recent study described two distinct circuits involved in fluid satiation: one controlling the satiation signal (involving MnPO^GLP1r^ and SFO^GLP1r^ neurons) and one controlling reward‐related drinking (involving dopaminergic neurons in the nucleus accumbens) (Augustine et al., 2019). It is likely that the satiation signal from Oxtr^PBN^ neurons interacts with the thirst satiation circuit via projections to the MnPO (first neural circuit), whereas the highly caloric, palatable solutions engage SFO^GLP1r^ neurons (second neural circuit).

We observed differences in FOS immunoreactivity in other subdivisions of the LPBN after fluid intake, including the el LPBN for caloric solutions and the cl LPBN for sweet and palatable solutions, suggesting these subdivisions may play a role in fluid intake differences (in addition to the dl LPBN). We examined el LPBN neurons by activating CGRP^PBN^ neurons to investigate whether there was a difference in intake of higher caloric versus lower caloric solutions; however, CGRP^PBN^ activation suppressed both types of solutions similarly, suggesting a generalised inhibitory response. The cl LPBN may be part of an alternate food satiation pathway (Garfield et al., 2015; Shah et al., 2014), which likely corresponds to a reward‐related subdivision of the LPBN (Han et al., 2018).

The peptide, oxytocin, is typically anorexigenic, so would be expected to have the opposite effect on AgRP neurons on Ensure intake (Maejima et al., 2014). Oxytocin neurons in the paraventricular hypothalamic nucleus (PVH) innervate the arcuate nucleus and increase FOS immunoreactivity in AgRP^ARC^ neurons, suggesting an interaction between oxytocin and the arcuate nucleus. Intra‐arcuate oxytocin administration decreased food intake, suggesting that arcuate activation may partially mediate the anorexigenic effect of oxytocin, perhaps via the anorexigenic proopiomelanocortin (POMC) neurons (Maejima et al., 2014). Oxytocin neurons in the paraventricular hypothalamic nucleus have previously been shown to project to Oxtr^PBN^ neurons and attenuate fluid intake, but less robustly than Oxtr^PBN^ neuron activation itself (Ryan et al., 2017).

Activation of Oxtr^PBN^ neurons decreased anxiety‐like behaviour, suggesting mice are less anxious when fluid satiated. This indicates that the initial fluid suppression is not because of anxiety‐like behaviour and is consistent with recent research indicating that the thirst response is anxiety‐provoking and aversive (Allen et al., 2017). We did not observe any significant difference in side preference in the conditioned place preference, suggesting that Oxtr^PBN^ activation has neutral valence.

Future directions include a more detailed characterisation of Oxtr^PBN^ neurons by examining colocalisation with other neurotransmitters, peptides and receptors, particularly those involved in feeding and fluid intake. For example, a recent paper demonstrated a role for prodynorphin (Pdyn)‐expressing neurons in the LPBN in the inhibition of fluid and food intake. In situ hybridisation experiments revealed only a partial overlap of Oxtr and Pdyn neurons (about 23%), suggesting these are predominantly distinct neural populations (Kim et al., 2020). Pdyn^PBN^ neurons are involved in the mechanosensory monitoring of ingestion and negative feedback control on intake behaviours upon distension of the digestive tract, so perhaps the overlap with Oxtr^PBN^ neurons involves only neurons which regulate fluid intake.

Future research could confirm the inhibitory AgRP response results in an in vivo model and investigate other fluid‐ and food‐related nuclei and projections from the LPBN, such as the role of the cl LPBN in reward‐related feeding, and the role of Oxtr^PBN^ neuron projections to nuclei involved in regulating thirst satiation, such as the MnPO.

Overall, results from this research suggest that the fluid satiation signal elicited by activation of Oxtr^PBN^ neurons can be modulated by feeding signals in several ways: the rapid, initial satiation signal may be inhibited by AgRP from the arcuate nucleus (Beutler et al., 2017), whereas more prolonged fluid‐satiation responses may also be inhibited by AgRP or alternatively by other reward‐related signals, likely via dopamine‐related signals which are independent of fluid satiation signals (Augustine et al., 2019). Such interactions between feeding‐ and fluid‐related signals may explain why sugary and alcoholic beverages are often consumed even after fluid‐satiation requirements are met.

## AUTHOR CONTRIBUTIONS

**Connor M. Aitken:** Data curation; formal analysis; investigation; writing – original draft. **Janine C. M. Jaramillo:** Data curation; formal analysis; investigation. **Warren Davis:** Data curation; formal analysis; investigation. **Liam Brennan‐Xie:** Data curation; formal analysis; investigation. **Stuart J. McDougall:** Data curation; formal analysis; investigation; methodology; writing – original draft. **Andrew J. Lawrence:** Resources; supervision. **Philip J. Ryan:** Conceptualization; data curation; formal analysis; funding acquisition; investigation; methodology; project administration; resources; supervision; writing – original draft; writing – review and editing.

## CONFLICT OF INTEREST STATEMENT

Andrew J. Lawrence is the current Editor‐in‐Chief of the *Journal of Neurochemistry*. The authors declare no competing financial conflict of interest.

### PEER REVIEW

The peer review history for this article is available at https://www.webofscience.com/api/gateway/wos/peer‐review/10.1111/jnc.15991.

## Supporting information


Table S1
Table S2
Table S3
Figure S1
Figure S2
Figure S3
Figure S4
Figure S5

## Data Availability

The data that support the findings of this study are available upon reasonable request.
