# Supplementary material for: Feeding signals inhibit fluid‐satiation signals in the mouse lateral parabrachial nucleus to increase intake of highly palatable, caloric solutions
Source: J Neurochem. 2023 Oct 19;167(5):648–67. doi: 10.1111/jnc.15991 (PMC10952698; doi:10.1111/jnc.15991)
Supplement: Supplementary file 1 — Table S1 Table S2 Table S3 Figure S1 Figure S2 Figure S3 Figure S4 Figure S5 [file JNC-167-648-s001.pdf]

SUPPLEMENTARY INFORMATION FOR:

FEEDING SIGNALS INHIBIT FLUID-SATIATION SIGNALS IN THE MOUSE LATERAL PARABRACHIAL NUCLEUS TO INCREASE INTAKE OF HIGHLY PALATABLE, CALORIC SOLUTIONS

Connor M Aitken[1,2], Janine C M Jaramillo[1,2], Warren Davis[1,2], Liam Brennan-Xie[1], Stuart J McDougall[1], Andrew J Lawrence[1,2], Philip J Ryan[1,2]

1. Florey Institute of Neuroscience & Mental Health, University of Melbourne, Parkville, VIC, 3052 Australia

2. Florey Department of Neuroscience & Mental Health, University of Melbourne, Parkville, VIC, 3052, Australia

Supplementary Table 1 Cohorts of mice for fluid intake experiments with ages and sexes.

Note that the same mice were used for the same solutions (at different concentrations); the only exception was for the mice given sucrose solutions, which were also given the saccharin solution.

Solutions	Number of cohorts	Total hM3	Total tdT	Av age (wk) for each cohort	Av weight (g) for each cohort
Ethanol	3	5M, 2F	6M, 4F	13.4 (1)<br>20.4 (2)<br>9.6 (3)	21.8 (1)<br>28.2 (2)<br>21.0 (3)
Sucrose and saccharin	2	4M, 1F	5M, 1F	24.3 (1)<br>18.2 (2)	30.9 (1)<br>27.6 (2)
Ensure®	3	5M, 4F	2M, 4F	17.6 (1)<br>9.9 (2)<br>9.5 (3)	27.8 (1)<br>20.9 (2)<br>20.7 (3)
Additional saccharin	2	2M, 3F	2M, 5F	10.4 (1)<br>9.2 (2)	22.5 (1)<br>19.0 (2)
Saline	2	3M, 3F	3M, 3F	14.8 (1)<br>10.2 (2)	26.8 (1)<br>22.9 (2)

Supplementary Table 2 Statistical results for Ensure® and paired water intake

	**15-min Ensure®**	**15-min water**	**2-h Ensure®**	**2-h water**
**1 kcal/ml**	$F(1,13) = 6.808$<br>$p = 0.0216$	$F(1,13) = 1.341$<br>$p = 0.2677$	$F(1,13) = 1.444$<br>$p = 0.2510$	$F(1,13) = 0.0087$<br>$p = 0.9721$
**0.6 kcal/ml**	$F(1,13) = 14.03$<br>$p = 0.0024$	$F(1,13) = 0.0365$<br>$p = 0.8514$	$F(1,13) = 4.826$<br>$p = 0.0468$	$F(1,13) = 0.5385$<br>$p = 0.4761$
**0.06 kcal/ml**	$F(1,13) = 35.48$<br>$p < 0.0001$	$F(1,13) = 8.153$<br>$p = 0.0135$	$F(1,13) = 3.042$<br>$p = 1.047$	$F(1,13) = 2.439$<br>$p = 0.1408$
**0.01 kcal/ml**	$F(1,13) = 21.18$<br>$p = 0.0005$	$F(1,13) = 2.826$<br>$p = 0.1166$	$F(1,13) = 26.44$<br>$p = 0.0002$	$F(1,13) = 3.373$<br>$p = 0.0892$

Supplementary Table 3 Statistical results for sucrose, ethanol, saccharin and saline and paired water

	15-min intake	15-min paired water	2-h intake	2-h paired water
**30% w/v sucrose**	F(1,9) = 9.048<br>p = 0.0148	F(1,9) = 3.934<br>p = 0.0786	F(1,9) = 2.982<br>p = 0.1183	F(1,9) = 22.25<br>p = 0.0011
**15% w/v sucrose**	F(1,9) = 6.734<br>p = 0.0290	F(1,9) = 0.01297<br>p = 0.9118	F(1,9) = 0.1958<br>p = 0.6686	F(1,9) = 0.0027<br>p = 0.9594
**1.5% w/v sucrose**	F(1,9) = 19.86<br>p = 0.0016	F(1,9) = 1.796<br>p = 0.2131	F(1,9) = 7.788<br>p = 0.0210	F(1,9) = 0.1056<br>p = 0.7527
**0.15% w/v sucrose**	F(1,9) = 9.717<br>p = 0.0124	F(1,9) = 2.982<br>p = 0.1183	F(1,9) = 47.45<br>p < 0.0001	F(1,9) = 3.379<br>p = 0.0852
**10% v/v ethanol**	F(1,15) = 21.12<br>p = 0.0004	F(1,15) = 3.034<br>p = 0.1020	F(1,15) = 1.372<br>p = 0.2597	F(1,15) = 0.647<br>p = 0.8027
**1% v/v ethanol**	F(1,15) = 6.957<br>p = 0.0186	F(1,15) = 3.378<br>p = 0.0860	F(1,15) = 3.618<br>p = 0.0765	F(1,15) = 0.1090<br>p = 0.3131
**0.1% v/v ethanol**	F(1,15) = 18.33<br>p = 0.0007	F(1,15) = 5.907<br>p = 0.0281	F(1,15) = 9.327<br>p = 0.0080	F(1,15) = 0.2529<br>p = 0.6224
**0.1% w/v saccharin**	F(1,21) = 9.563<br>p = 0.0055	F(1,21) = 0.1332<br>p = 0.7188	F(1,21) = 0.1800<br>p = 0.6757	F(1,21) = 0.4844<br>p = 0.4941
**0.15M saline**	F(1,10) = 1.639<br>p = 0.2294	F(1,10) = 6.176<br>p = 0.0323	F(1,10) = 0.6688<br>p = 0.4325	F(1,10) = 3.431<br>p = 0.0937
**0.3M saline**	F(1,10) = 2.152<br>p = 0.1731	F(1,10) = 10.36<br>p = 0.0092	F(1,10) = 0.4613<br>p = 0.5124	F(1,10) = 5.997<br>p = 0.0343
**0.5M saline**	F(1,10) = 20.75<br>p = 0.0010	F(1,10) = 4.999<br>p = 0.0493	F(1,10) = 12.22<br>p = 0.0058	F(1,10) = 8.925<br>p = 0.0136

A. RAPID INITIAL INTAKE (15 min)

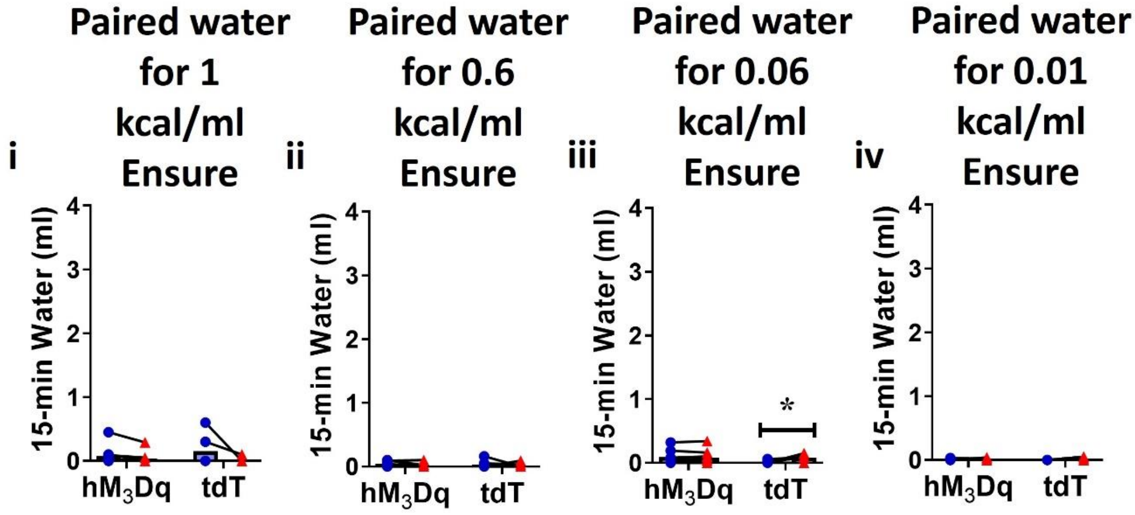

B. CUMULATIVE INTAKE (2 h)

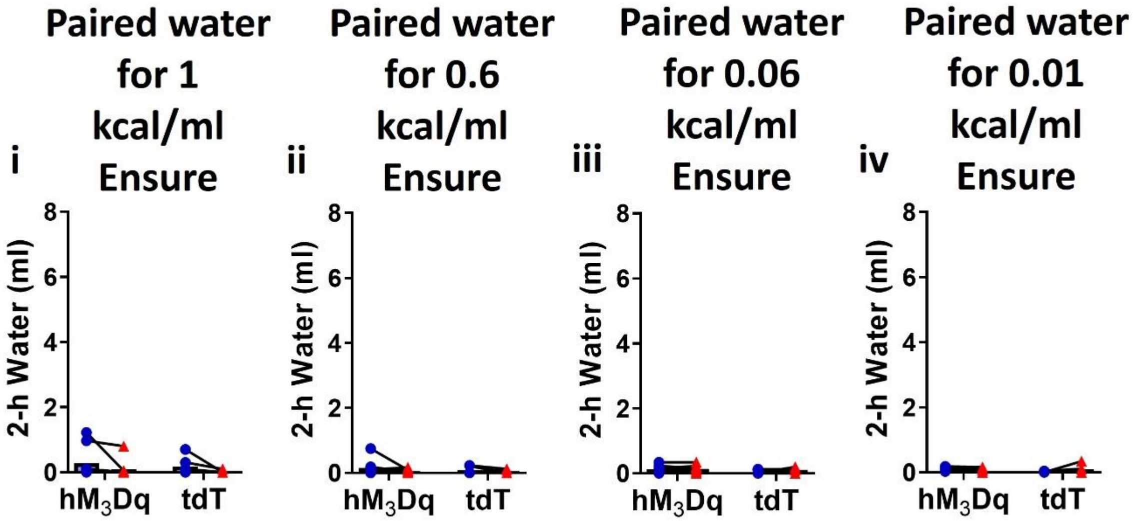

Supplementary Figure 1. Activation of Oxtr^{PBN} had no significant effect on paired water in the Ensure® two-bottle choice experiments. ***A.*** i-iv) Oxtr^{PBN} stimulation had no significant difference in 15-min (rapid) paired water intake (except for paired water with 0.06 kcal/ml Ensure® where water intake was low). ***B.*** i-iv) Oxtr^{PBN} stimulation had no significant difference in 2-h (cumulative) paired water intake. Data expressed as mean ± s.e.m; n = 9 hM$_3$Dq, 6 tdTomato; ****$p < 0.0001$; ***$p < 0.001$; *$p < 0.05$.

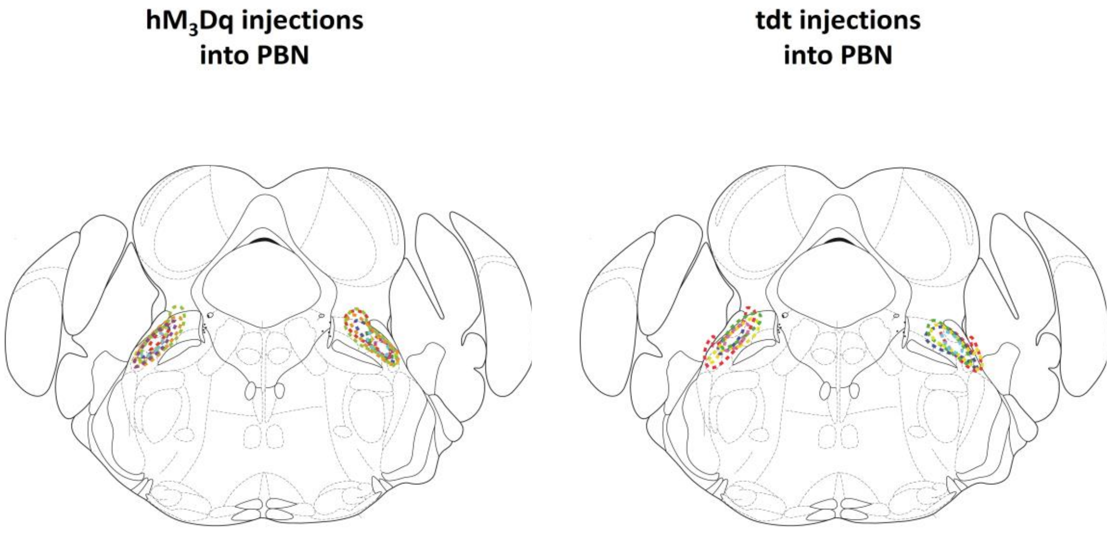

Supplementary Figure 2. Schematic representations of the spread of the virus in hM$_3$Dq- and tdT-injected mice (Paxinos & Franklin 2013). Each mouse within the cohort is represented by a different colour.

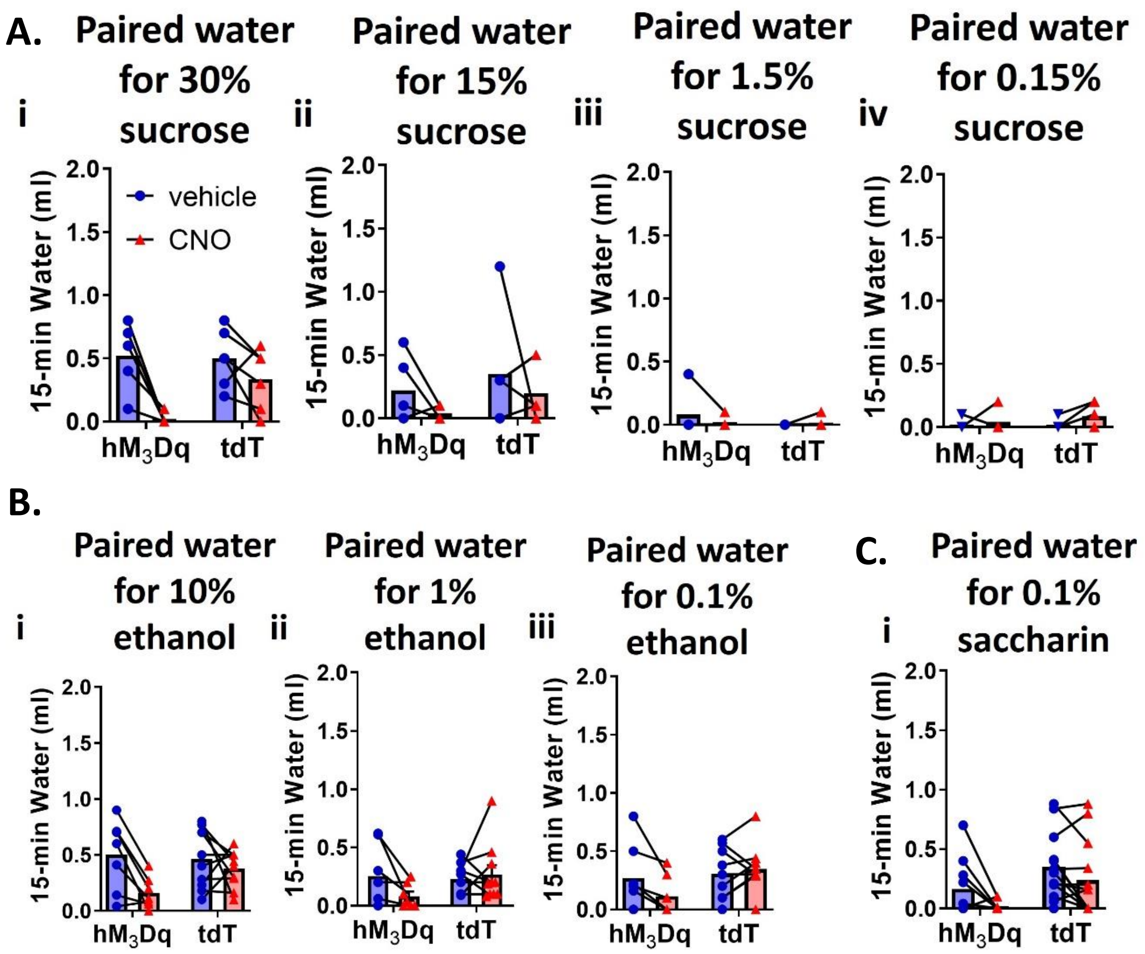

Supplementary Figure 3. 15-min paired water intake was not significantly different, except for a significant interaction for paired water intake for 0.1% ethanol. ***A, i-iv)*** Paired water intake for sucrose was not significantly different; n = 5 hM_3Dq, 6 tdTomato. ***B, i-iii)*** Paired water intake for ethanol was not significantly different for 10% or 1% ethanol, but there was a significant interaction for paired water for 0.1% v/v ethanol ($F(1,15) = 9.33$; $p = 0.03$); n = 7 hM_3Dq, 10 tdTomato. ***C, i)*** Paired water intake for saccharin was not significantly different; n = 10 hM_3Dq, 13 tdTomato. Data expressed as mean ± s.e.m.

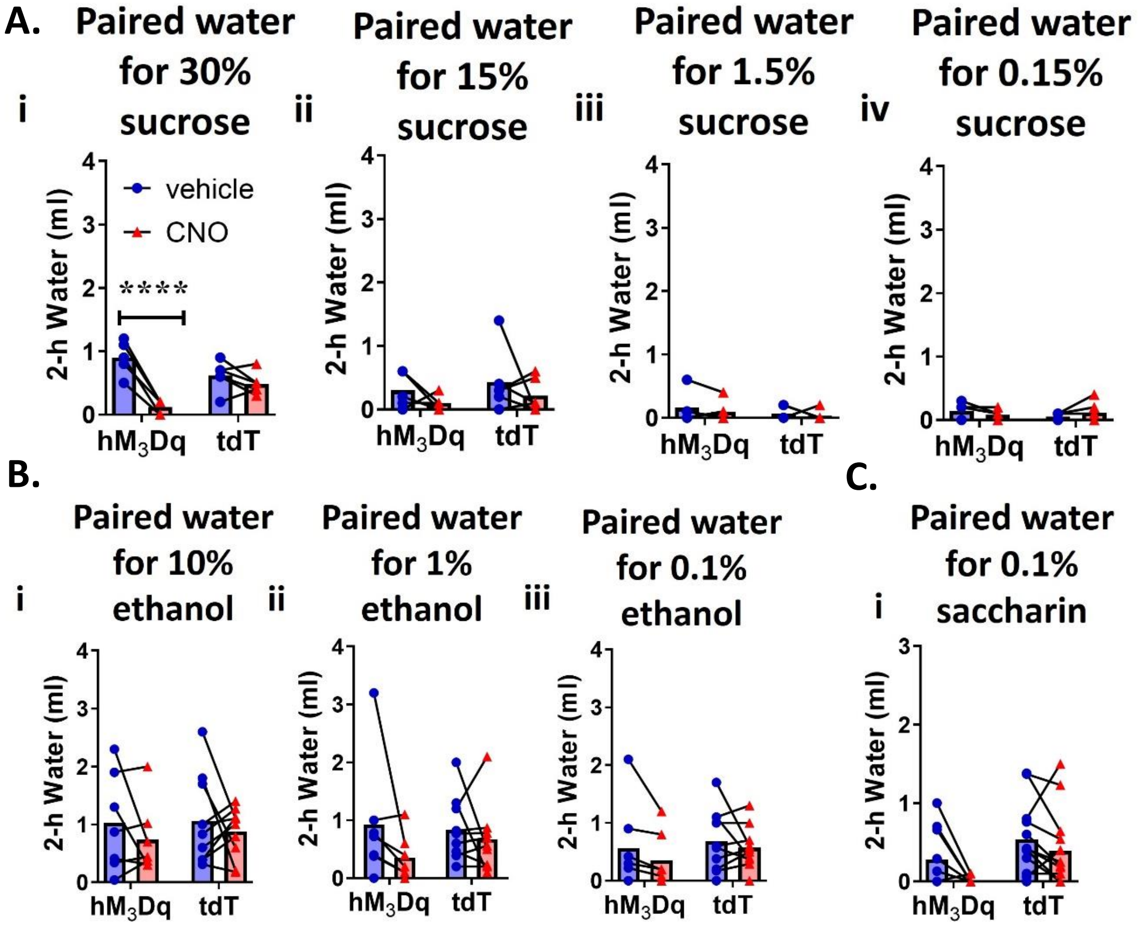

Supplementary Figure 4. 2-h paired water intake was not significantly different, except for a significant difference for paired water intake for 30% sucrose. ***A, i-iv)*** Paired water intake for sucrose was not significantly different, except for 30% w/v sucrose; n = 5 hM$_3$Dq, 6 tdTomato. ***B, i-iii)*** Paired water intake for ethanol was not significantly different; n = 7 hM$_3$Dq, 10 tdTomato. ***C, i)*** Paired water intake for saccharin was not significantly different; n = 10 hM$_3$Dq, 13 tdTomato. Data expressed as mean ± s.e.m; ****$p<0.0001$.

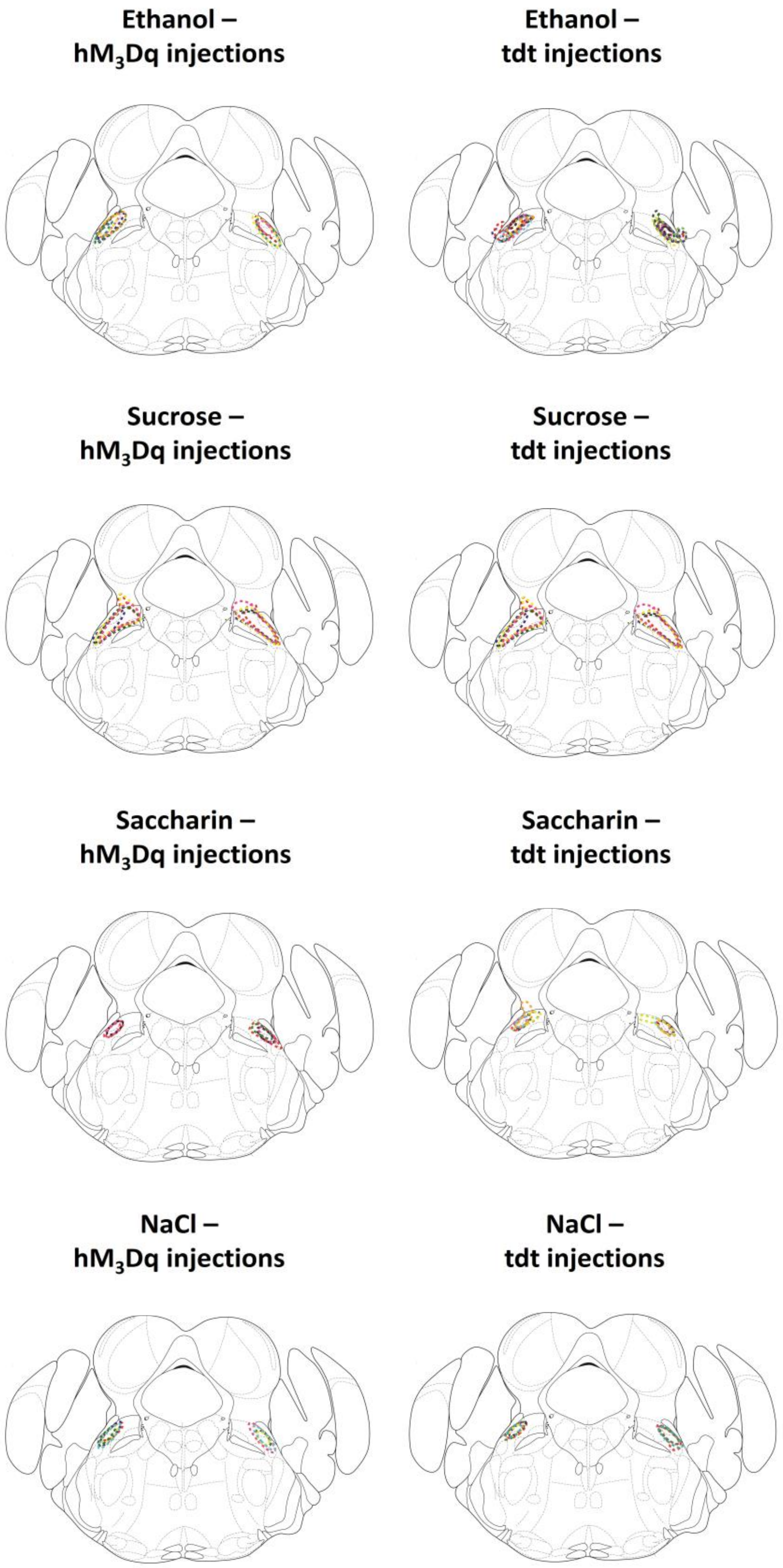

Supplementary Figure 5. Schematic representations of the spread of the virus in hM_3Dq- and tdT-injected mice (Paxinos & Franklin 2013). Each mouse within each cohort is represented by a different colour.
